# Food restriction increase the expression of mTORC1 complex genes in the skeletal muscle of juvenile pacu (*Piaractus mesopotamicus*)

**DOI:** 10.1371/journal.pone.0177679

**Published:** 2017-05-15

**Authors:** Tassiana Gutierrez de Paula, Bruna Tereza Thomazini Zanella, Bruno Evaristo de Almeida Fantinatti, Leonardo Nazário de Moraes, Bruno Oliveira da Silva Duran, Caroline Bredariol de Oliveira, Rondinelle Artur Simões Salomão, Rafaela Nunes da Silva, Carlos Roberto Padovani, Vander Bruno dos Santos, Edson Assunção Mareco, Robson Francisco Carvalho, Maeli Dal-Pai-Silva

**Affiliations:** 1 Department of Morphology, Institute of Bioscience of Botucatu, São Paulo State University, Botucatu, São Paulo, Brazil; 2 Aquaculture Center, São Paulo State University, Jaboticabal, São Paulo, Brazil; 3 Department of Biostatistics, Institute of Bioscience of Botucatu, São Paulo State University, Botucatu, São Paulo, Brazil; 4 São Paulo Agency for Agribusiness Technology, Presidente Prudente, São Paulo, Brazil; 5 University of Western São Paulo (UNOESTE), Presidente Prudente, São Paulo, Brazil; Universidad Pablo de Olavide, SPAIN

## Abstract

Skeletal muscle is capable of phenotypic adaptation to environmental factors, such as nutrient availability, by altering the balance between muscle catabolism and anabolism that in turn coordinates muscle growth. Small noncoding RNAs, known as microRNAs (miRNAs), repress the expression of target mRNAs, and many studies have demonstrated that miRNAs regulate the mRNAs of catabolic and anabolic genes. We evaluated muscle morphology, gene expression of components involved in catabolism, anabolism and energetic metabolism and miRNAs expression in both the fast and slow muscle of juvenile pacu (*Piaractus mesopotamicus*) during food restriction and refeeding. Our analysis revealed that short periods of food restriction followed by refeeding predominantly affected fast muscle, with changes in muscle fiber diameter and miRNAs expression. There was an increase in the mRNA levels of catabolic pathways components (*FBXO25*, *ATG12*, *BCL2*) and energetic metabolism-related genes (*PGC1α* and *SDHA*), together with a decrease in *PPARβ/δ* mRNA levels. Interestingly, an increase in mRNA levels of anabolic genes (*PI3K* and *mTORC1* complex: *mTOR*, *mLST8* and *RAPTOR*) was also observed during food restriction. After refeeding, muscle morphology showed similar patterns of the control group; the majority of genes were slightly up- or down-regulated in fast and slow muscle, respectively; the levels of all miRNAs increased in fast muscle and some of them decreased in slow muscle. Our findings demonstrated that a short period of food restriction in juvenile pacu had a considerable impact on fast muscle, increasing the expression of anabolic (*PI3K* and *mTORC1* complex: *mTOR*, *mLST8* and *RAPTOR*) and energetic metabolism genes. The miRNAs (miR-1, miR-206, miR-199 and miR-23a) were more expressed during refeeding and while their target genes (*IGF-1*, *mTOR*, *PGC1α* and *MAFbx*), presented a decreased expression. The alterations in *mTORC1* complex observed during fasting may have influenced the rates of protein synthesis by using amino acids from protein degradation as an alternative mechanism to preserve muscle phenotype and metabolic demand maintenance.

## Introduction

The pacu is a freshwater neotropical characid fish characterized by hardiness, fast growth, adaptation to artificial feeding and tasty meat [1] that has a high market value to Brazilian fisheries and pisciculture [2]. Muscle growth in fish is a complex process controlled by a dynamic balance of anabolic and catabolic molecular pathways and is influenced by several intrinsic and extrinsic factors, such as food availability, water quality, temperature, developmental stage and geographic distribution [3,4]. During conditions that promote the loss of muscle mass, such as food restriction, an increase in the activity of the proteolytic ubiquitin-proteasome system, particularly the ubiquitin-ligases [5], and the autophagy pathway occurs [6]. These events promote increased rates of protein degradation, leading to muscle atrophy [5,6].

Although numerous ubiquitin-ligases have been identified, *FBXO25* (*F-Box Protein 25/ubiquitin protein*), *MURF1* (*Muscle Ring Finger protein-1*) and *MAFbx* (*Muscle Atrophy F-box* or *atrogin*), also known as atrogenes, are E3 ubiquitin-ligase that are up-regulated during increased muscle catabolic activity [7], such as food restriction conditions in fish [4,8–10]. Additionally, atrogenes play a critical role in controlling protein turnover in skeletal muscle to maintain muscle function [11,12].

The major anabolic process responsible for the increase in muscle protein synthesis in mammals and in fish is controlled by *IGF-I* (*insulin-like growth factor-I*) [13]. The *IGF-I* pathway has an important role in the inhibition of muscle protein degradation by blocking the up-regulation of the E3 ubiquitin ligases *MURF1* and *MAFbx* [8].

Studies using mammals have shown that the activation of *mTOR*, a component of two different complexes, *mTORC1* (comprising *mTOR*, *mLST8*, and *RAPTOR*) and *mTORC2* (comprising *mTOR*, *mLST8*, and *RICTOR*) [14–16], ultimately results in the up regulation of key genes that induce muscle mass gain [13–15,17–19].

The anabolic and catabolic signaling pathways of skeletal muscle are controlled by small noncoding RNAs known as microRNAs (miRNAs), and several studies have shown that the miRNAs -1, -206, -23a and -199 inhibit genes that stimulate and repress muscle development and growth [20–22]. Additionally, miR-1, -199 and -206 were shown to control the *IGF-1* gene since its expression levels were inversely proportional to those of the miRNAs [22–24], and miR-23a regulates the expression of *PGC-1a*, an important cofactor of mitochondrial biogenesis, and *MAFbx*, an atrogene with a crucial role in protein degradation [25–27].

Pacu (*Piaractus mesopotamicus*) is a tropical fast-growing fish that can attain a large body size [1], and this species is of commercial interest for aquaculture. With the goal to understand the intracellular signaling pathways involved in the regulation of muscle growth in fish, *in vivo* studies have evaluated the impact of feeding/refeeding protocols on muscle growth-related genes, as these protocols promote changes in the homeostasis between muscle catabolic and anabolic states. Additionally, it is unclear whether muscle protein synthesis and degradation operate independently or whether these processes can act together in the control of muscle mass during food restriction/refeeding. Using this approach, we evaluated muscle morphology, mRNA expression of components involved in catabolism, anabolism and energetic metabolism and miRNA expression in both fast and slow muscle of juvenile pacu during food restriction and refeeding.

## Material and methods

### Ethics statement

All experiments and procedures were carried out in accordance with the Ethical Principles in Animal Research adopted by the Brazilian College of Animal Experimentation (COBEA). This protocol was approved by the Ethics Committee on Animal Use (protocol number 694-CEUA-Ethics Commission on the use of animals) of the Institute of Biosciences of Botucatu, São Paulo State University, Botucatu, São Paulo, Brazil. Animals were euthanized with benzocaine at a concentration exceeding 250 mg/L prior to the collection of muscle samples.

### Sample collection

Pacu fish (*Piaractus mesopotamicus*) were obtained from the Sao Paulo Agency for Agribusiness Technology (APTA), Presidente Prudente, Sao Paulo, Brazil. Juvenile fish (approximately 150 g) were farmed at 28°C under a natural photoperiod (12 hours of light: 12 hours of dark) in storage tanks of 0.5 m^3^ equipped with separate systems of water circulation. Fish were acclimatized for 1 week under satiety feeding conditions. At the beginning of the experiment, food was withdrawn from the fish for 10 days (fasting condition) followed by 60 hours of refeeding. Samples of fast muscle were collected from the epaxial region, near the head, and slow muscle samples were collected near the lateral line. Muscle samples were collected at zero (Control group—C) and 10 days of fasting (Fasting group—F) and after 6 (Refeeding group—R6) and 60 hours of refeeding (Refeeding group—R60).

### Morphometric analysis

Fast and slow muscles samples were collected and quickly frozen in liquid nitrogen-cooled isopentane and stored at −80°C before sectioning. Muscle histological cryosections (10 μm) from the C, F and R60 groups were cut and stained using the hematoxylin-eosin (HE) method [28]. Muscle fiber diameters were determined by measuring 1000 fast and slow muscle fibers from each animal (8 animals) per group, using a compound microscope attached to a computerized imaging analysis system (Leica Qwin, Wetzlar, Germany) [29]. The fiber diameter (D) was estimated indirectly from individual fiber area (A) using the formula D = 2A 0.5 π-0.5 [30]. For each group, muscle fiber diameters were grouped into classes (<30; 30 ┤ 50; 50 ┤ 70; 70 ┤ 90 and >90 μm) based on Johnston [31]and de Almeida et al. [32]. Muscle fiber frequency in the classes corresponds to the number of fibers from each diameter class relative to the total number of fibers measured.

### Succinate dehydrogenase (SDH) analysis

SDH analysis was used as an indicator of muscle fiber oxidative capacity in fast and slow muscle and was performed as described by Nachlas et al.[33]. Transverse cryosections (10 μm) of muscle from the C, F and R60 groups were placed on the same slide to minimize staining differences. The cryosections were incubated with 5 ml of 0.2 M sodium succinate solution and 10 ml aqueous solution of nitro blue tetrazolium (NBT, 1 mg/ml). The samples were incubated for 20 to 30 minutes at 37°C, washed in saline, fixed subsequently in formol saline for 10 minutes, rinsed in 15% ethanol for four/five minutes and mounted in Permount. To analyze SDH activity, images of all samples were captured using a microscope (40X magnification) attached to a computerized imaging analysis system. The light intensity and filter alignment parameters used were the same for all samples. Quantitative analysis of SDH staining intensity was determined by measuring the background staining (gray scale) with Image Analysis System Software (Leica Qwin, Germany).

### Gene expression analysis of mRNAs and miRNAs involved in anabolic and catabolic processes

Total RNA was extracted from fast and slow muscle samples in the C, F, R6 and R60 groups for mRNA analysis using TRIzol^®^ Reagent (Life Technologies, USA), according to the manufacturer’s recommendations. The RNA quantification was performed using the spectrophotometer NanoVue^™^ Plus (GE Healthcare, USA), which also determined the RNA purity by measuring the absorbance at 260 nm (RNA quantity) and 280 nm (protein quantity). Only samples with 260/280 ratio ≥ 1.8 were used. The RNA integrity was evaluated through capillary electrophoresis in the 2100 Bioanalyzer (Agilent, USA), which provided a RNA integrity number (RIN) based on the *28s* and *18s* ribosomal RNAs. Only samples with RIN ≥ 7.0 were used. Extracted RNA was treated with DNase I Amplification Grade (Life Technologies, USA) to eliminate any possible contamination with genomic DNA from the samples. mRNA reverse transcription was performed using the GoScript^™^ Reverse Transcription System (Promega, USA), following the manufacturer’s guidelines. miRNA expression was assessed in the C, F and R60 groups using a TaqMan^®^ MicroRNA Reverse Transcription kit (Life Technologies, USA) combined with TaqMan^®^ MicroRNA Assays (Life Technologies, USA), according to the protocol instructions.

The expression levels of miRNAs and mRNAs were assessed by quantitative real-time PCR (qPCR) using the QuantStudio^™^ 12K Flex Real-Time PCR System (Life Technologies, USA). Each cDNA sample corresponding to a miRNA was amplified by TaqMan^®^ Universal PCR Master Mix (Life Technologies, USA) and TaqMan^®^ MicroRNA Assays (Life Technologies, USA), which contain primers and specific probes to miR-1, miR-199, miR-23a, miR-206 and U6 snRNA (endogenous control) (S1 Table, S1 File). The cDNA samples corresponding to the mRNA of the genes analyzed were amplified by GoTaq^®^ qPCR Master Mix (Promega, USA), and primers were synthesized by Life Technologies (USA), which were designed using Primer Express 3.0.1^*®*^ (Life Technologies, USA) (S2 Table). The expression levels were normalized by *GAPDH*, whose expression was constant among all samples. The relative quantification of gene expression was performed by the comparative Ct method [34] using Data Assist 2.0 (Life Technologies).

### Heat map summary of clustering of catabolic, anabolic and energetic metabolism data

To establish relationships among all the components of the signaling pathways studied, a heat map summary and hierarchical clustering analysis were performed using the Ct data and the R Bioconductor packages gplots (version 3.0.1) and heatmap.2 (version 3.0.1). Clustering and seriation were based on Pearson’s correlation coefficient of z-score normalized abundance values (scaled from 0 to 1).

### Statistical analysis

Muscle fiber diameter data are expressed as frequency percentage and were analyzed by a Goodman test between and within multinomial population [35]. The SDH evaluation and mRNA and miRNA relative expression were analyzed by Kruskal-Wallis Test followed by Dunn’s multiple comparisons test [36]. Statistical significance was set at P<0.05 for all analyses.

## Results

### Morphometric analysis

**Fig 1:**
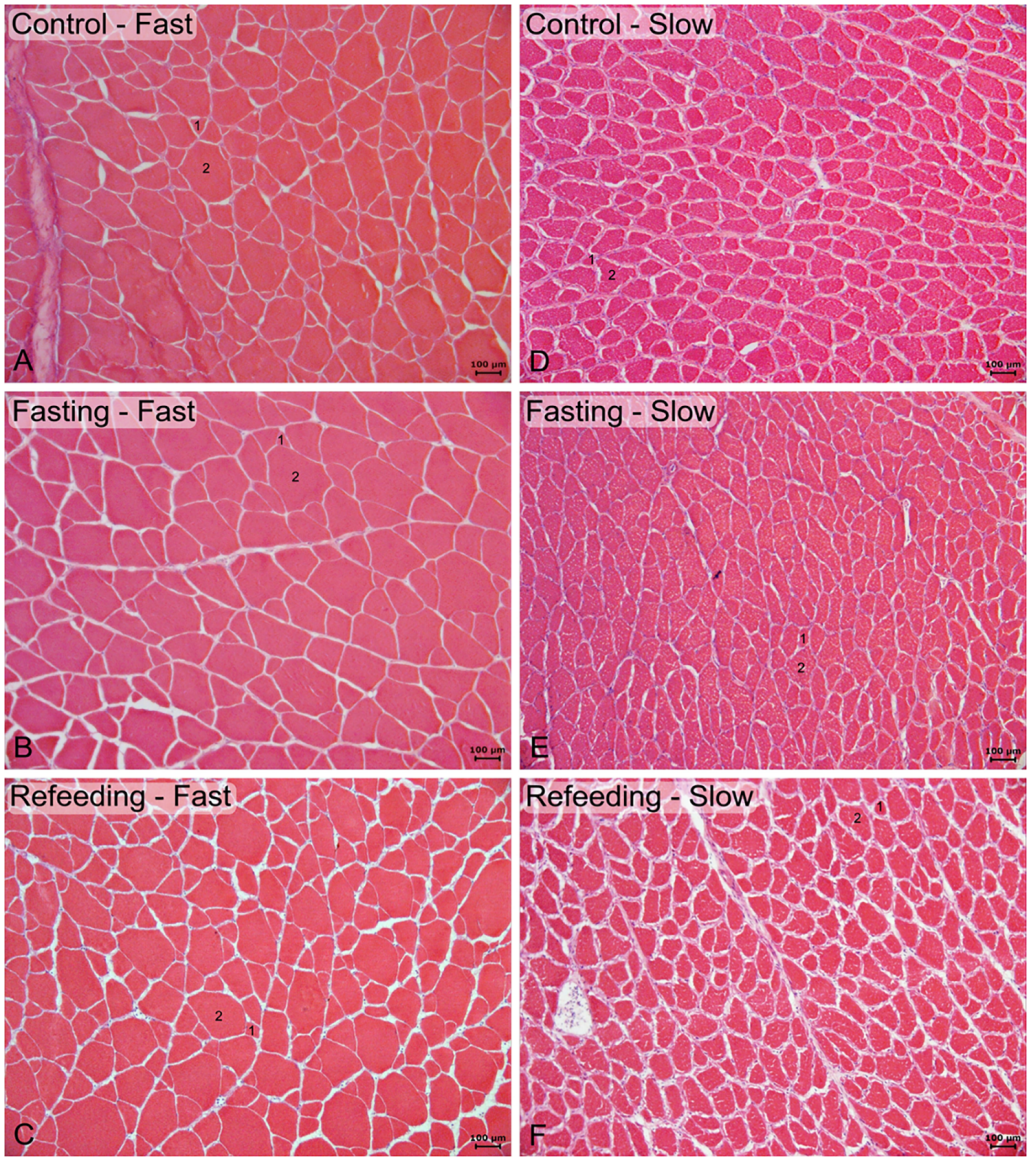
Hematoxylin and eosin (HE) staining showing fast muscle (left side) and slow muscle (right side). A and D: Control (C) group. B and E: Fasting (F) group. C and F: Refeeding (R60) group. Round and polygonal fibers distributed in a mosaic pattern characterized by small (1) and large fibers (2). Scale bar: 100 μm.

**Fig 2:**
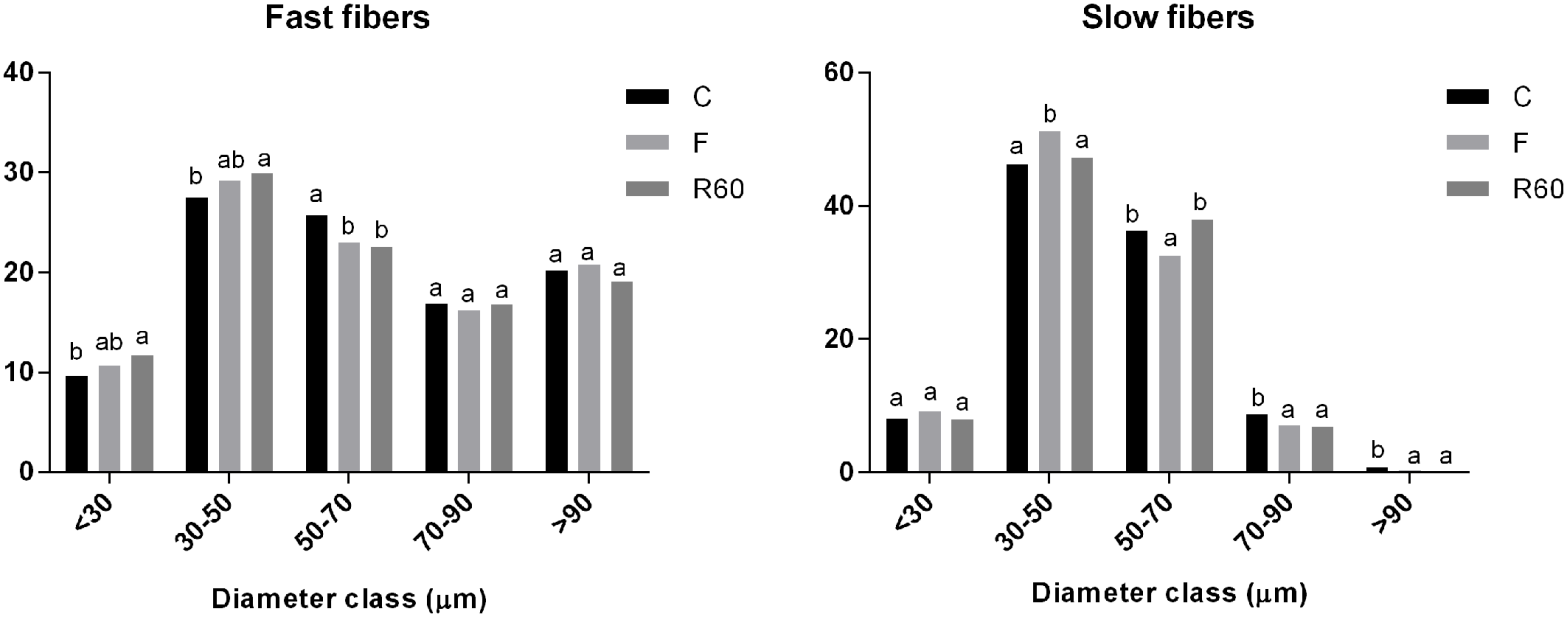
Frequency distribution of fast and slow muscle fibers of pacu juveniles. Control, before fasting (C); after 10 days of fasting (F) and after 60 hours of refeeding (R60). Letters in the columns compare the frequency of fibers between the groups. Values with the same letters are not statistically significant between the periods (P <0.05. Goodman test).

The muscle morphology of fast and slow muscle showed round and polygonal fibers distributed in a mosaic pattern characterized by fibers of different diameters (Fig 1). Comparing to the C group, during food restriction, there were a decrease in the frequency of fast muscle fiber diameter in the 50–70 μm; slow muscle presented a decrease in muscle fiber diameter in the classes from 50 to 90 μm and an increase in fiber frequency in the 30–50 μm class occured. After the refeeding period, only slow muscle showed increases in the frequency of fibers in the 50–70 μm class compared with the F group (Fig 2).

### Succinate dehydrogenase (SDH) analysis

**Fig 3:**
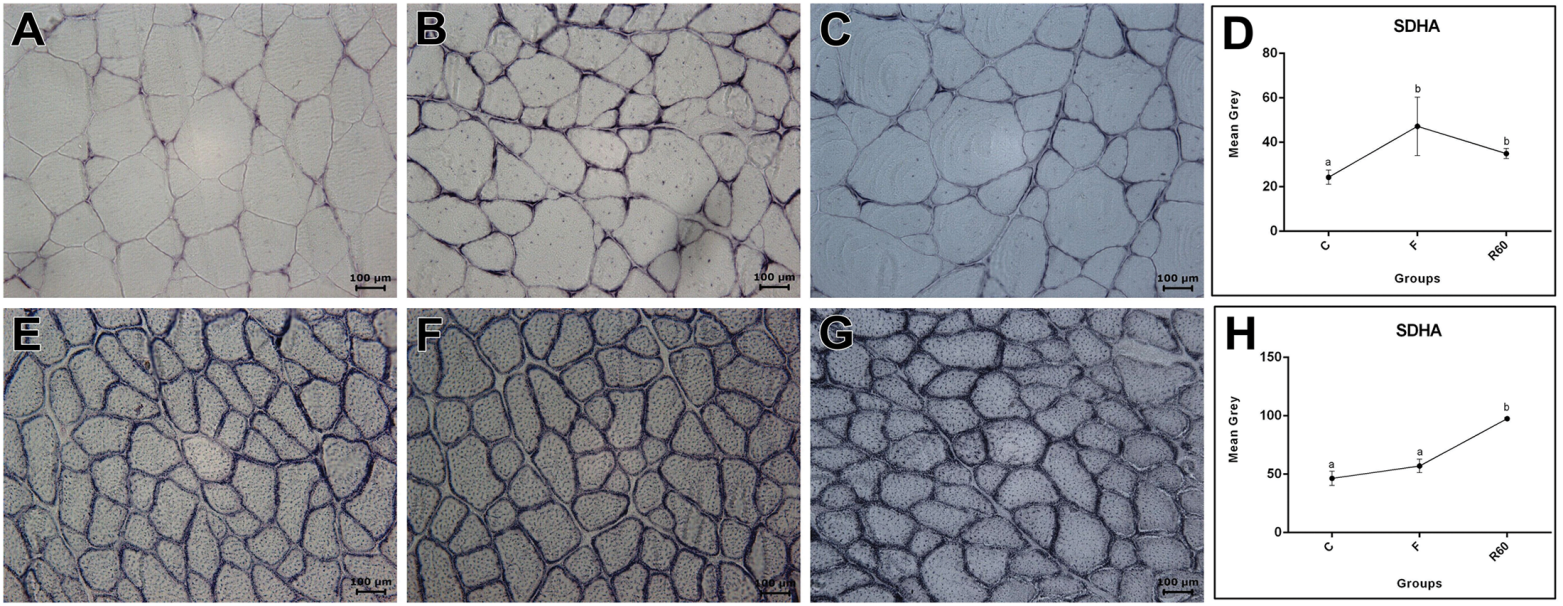
Succinate dehydrogenase (SDH) histochemistry reaction in fast (top) and slow (down) muscles. A and E: Control (C) group. B and F: Fasting (F) group. C and G: Refeeding (R60) group. Scale bar: 100 μm. Assessment of SDH activity in fast (D) and slow (H) muscle.

As a general indicator of muscle fiber oxidative capacity in both fast and slow muscles, we measured SDH activity. The results showed differences in SDH activity between fast and slow muscle (data not show) and among the groups evaluated. We observed an increase in SDH activity in fast muscle in F compared with the C and R60 groups. In slow muscle, SDH activity was higher in R60 than that of the C and F groups (Fig 3).

### Gene expression in fast and slow muscle: Fasting and refeeding periods

In fast muscle of pacu, a fasting period promoted an increase in the expression of the catabolic genes *ATG12* (*Related Autophagy 12/Ubiquitin-like*), *BCL2* (*B-cell CLL/Lymphoma 2*) and *FBXO25* (*F-Box Protein 25/ubiquitin protein*), the anabolic gene *PI3K* (*Phosphatidylinositol 3 Kinase*, *Catalytic Subunit Type 3*), and the *mTORC1* complex components, *mLST8* (*Target of rapamycin subunit complex LST8*), *mTOR* (*Target of Mechanistic Rapamycin*) and *RAPTOR* (*subunit complex mTORC1*), compared with the C group. The expression of genes related to energy metabolism, such as *SDHA* (*Succinate Dehydrogenase Complex*, *Subunit A*, *Flavoprotein*) and *PGC1α* (*Peroxisome proliferator-activated receptor gamma*, *coactivator 1*), was high compared with the C group, and the *PPARβ/δA* (*Peroxisome Proliferator-Activated Receptor Beta/Delta isoform*) level decreased compared with the C group.

**Fig 4:**
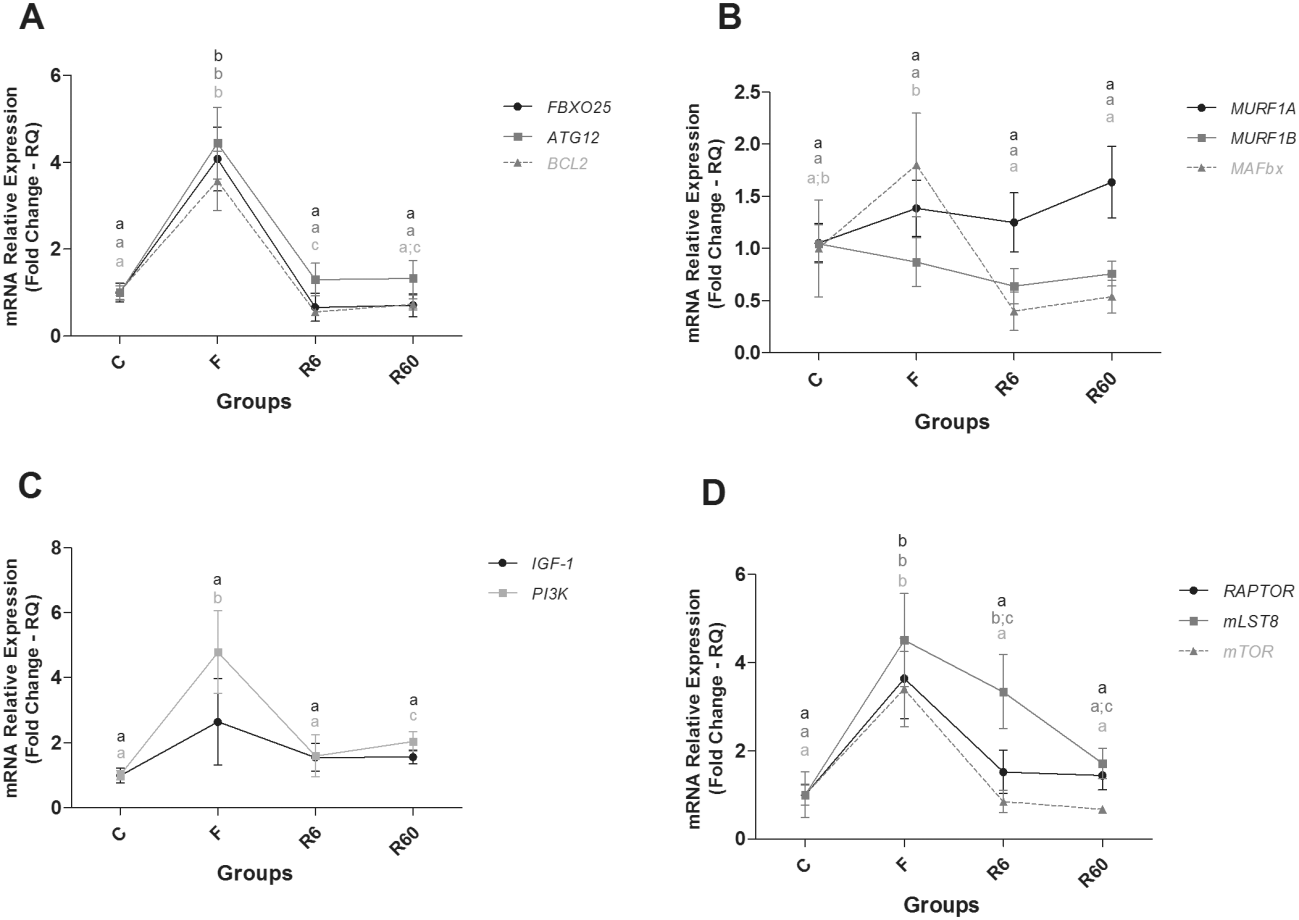
Relative mRNA expression of catabolic and anabolic pathway components in fast muscle. Groups C (control), F (fasting—10 days of fasting), R6 (6 hours of refeeding) and R60 (60 hours of refeeding). The data are expressed as fold change. Different letters indicate significant differences in expression between the groups (P < 0.05). The data are presented as the mean ± SEM (n = 8).

**Fig 5:**
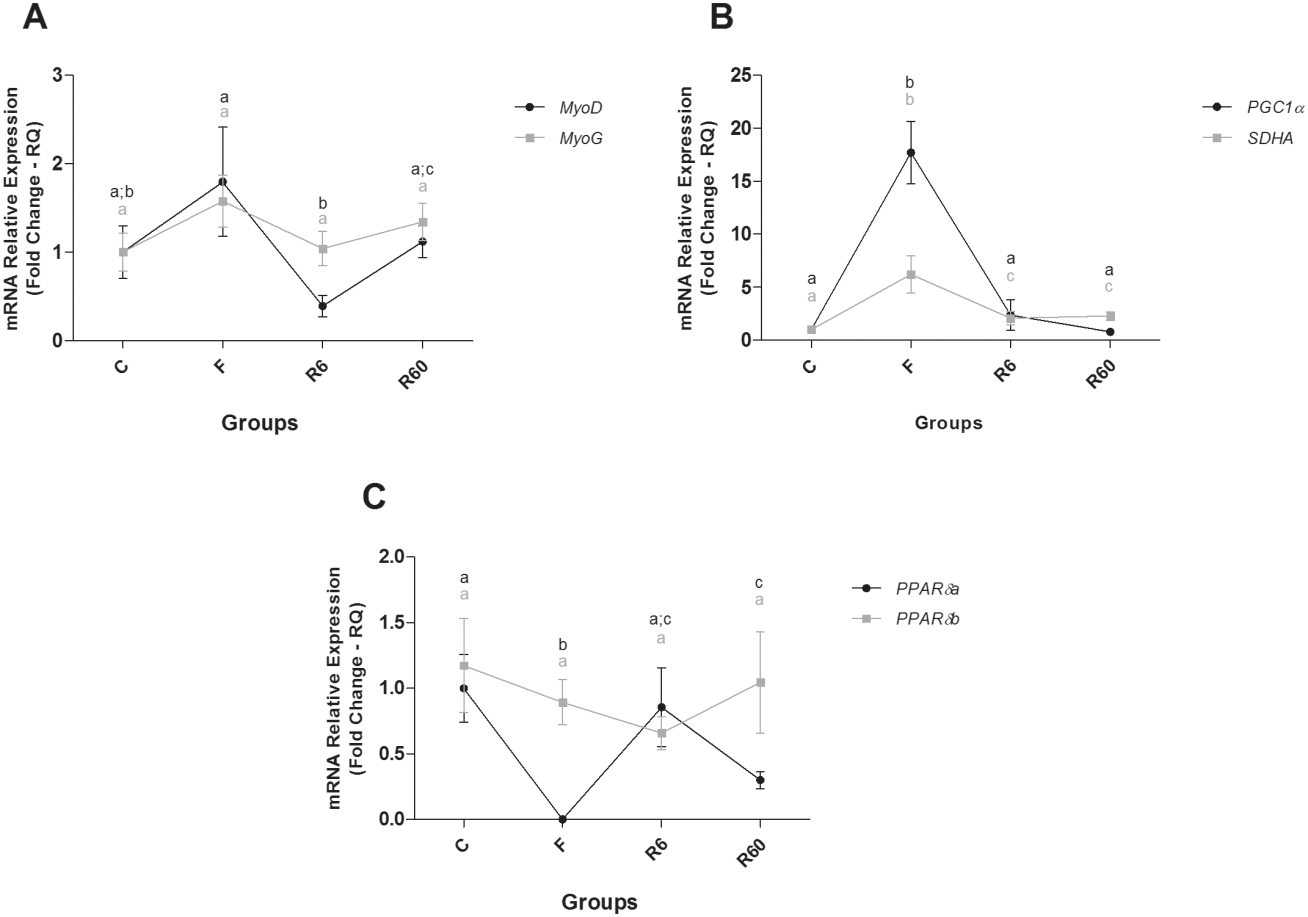
Relative mRNA expression of anabolic pathway and energetic metabolism components in fast muscle. Groups C (control), F (fasting—10 days of fasting), R6 (6 hours of refeeding) and R60 (60 hours of refeeding). The data are expressed as fold change. Different letters indicate significant differences in expression between the groups (P < 0.05). The data are presented as the mean ± SEM (n = 8).

After 6 and 60 hours of refeeding, most catabolic genes showed decreased expression levels compared with the F group, which was similar to the C group. R6 also showed decreased *BCL2* gene expression compared with the C group. Regarding anabolic genes, R6 had similar *PI3K* expression to that of the C group, which was lower than R60. The components of the *mTORC1* complex, *mTOR* and *RAPTOR*, were decreased compared with F, which was similar to the C group, whereas *mLST8* gene expression levels decreased gradually from the F to R60 groups. *SDHA* gene expression decreased in R6 and R60 compared with F and was high compared with the C group. *PGC1α* gene expression in R6 and R60 was decreased compared with F, and it was similar to the C group; *MyoD* gene expression decreased in R6 compared with F and R60 and *PPARβ/δA* gene expression increased during the refeeding period, with R6 being similar to C group and R60 lower than that of the C group (Figs 4 and 5).

**Fig 6:**
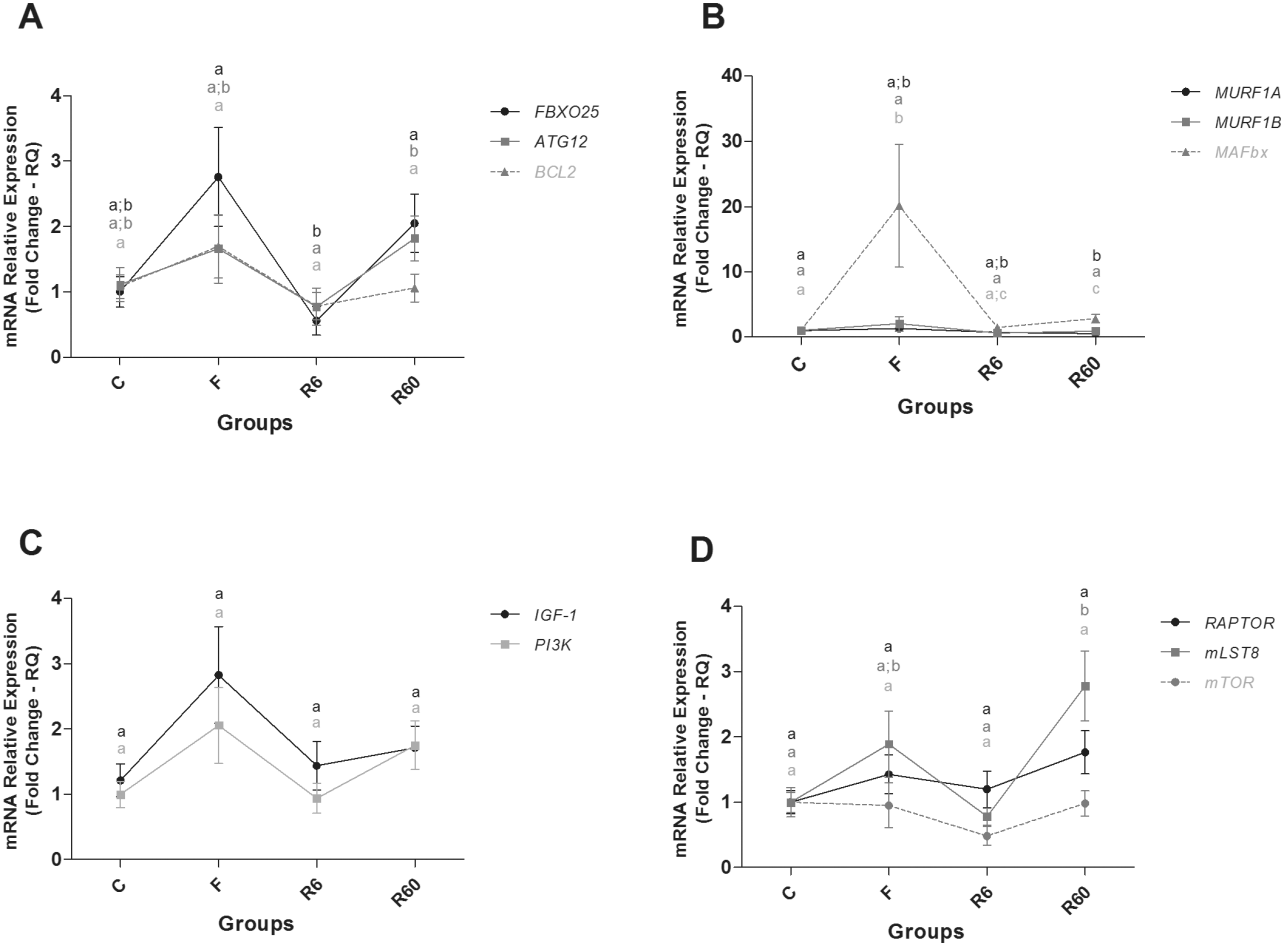
Relative mRNA expression of catabolic and anabolic pathway components in slow muscle. Groups C (control), F (fasting—10 days of fasting), R6 (6 hours of refeeding) and R60 (60 hours of refeeding). The data are expressed as fold change. Different letters indicate significant differences in expression between the groups (P < 0.05). The data are presented as the mean ± SEM (n = 8).

**Fig 7:**
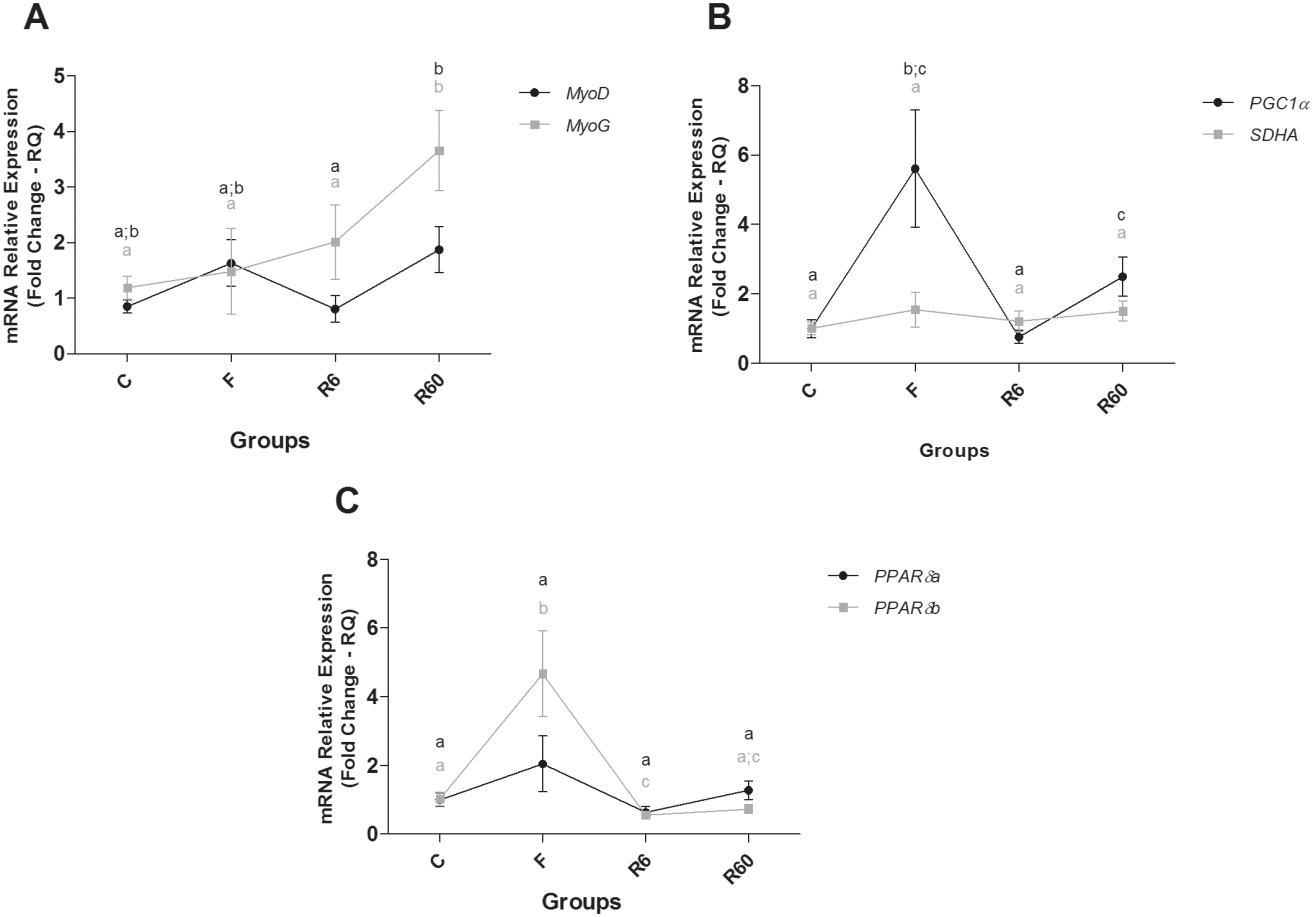
Relative mRNA expression of anabolic pathway and energetic metabolism components in slow muscle. Groups C (control), F (fasting—10 days of fasting), R6 (6 hours of refeeding) and R60 (60 hours of refeeding). The data are expressed as fold change. Different letters indicate significant differences in expression between the groups (P < 0.05). The data are presented as the mean ± SEM (n = 8).

In slow muscle of pacu, ten days of fasting promoted a slight change in gene expression. There was increased expression of the catabolic gene *MAFbx* (also called *FBOXO32* or *Atrogin-1*) and of genes related to energy metabolism, *PGC-1α* and *PPARβ/δB*, compared with the C group. After 6 hours of refeeding (R6), *MAFbx* gene expression decreased compared with F, and it was similar to the R60 group. *ATG12* gene expression was higher in R60 than that in the R6 group. *FBXO25* gene levels decreased in R6 compared with the F and R60 groups. *MURF1A* gene expression in R60 decreased in comparison to the C group. With regard to anabolic gene expression, only a component of the *mTORC1* complex, *mLST8*, was higher in the R60 group compared with the C group; *PGC1α* gene expression decreased in R6 compared with F and increased compared with the R60 group. *PPARβ/δB* gene expression was lower in R6 than in the C and F groups. *PPARβ/δB* gene expression in R6 was similar to R60, which was lower than that of the F group. After 60 hours (R60) of refeeding, the *MAFbx*, *mLST8*, *PGC1α*, and *MYOG* genes were significantly higher in R60 than in C group; the *MURF1A* gene was significantly lower in R60 than in C group. *MyoD* gene expression increased in R60 compared with R6, and *MYOG* gene expression increased in R60 compared with C, F and R6 groups. *PPARβ/δB* gene expression was significantly lower in R60 compared with the C group (Figs 6 and 7).

### miRNAs expression in fast and slow muscle: Fasting and refeeding period

In order to complement our experiment, we performed a comparison of the miRNA target sites at the 3’UTR of the examined mRNAs between different species of vertebrates (*Homo sapiens*, *Mus musculus*, *Danio rerio* and *Piaractus mesopotamicus*) using Geneious 4.8.5 software [37]. The comparisons revealed a considerable conservation of the 3’UTR nucleotide sequences between the species (S1 File).

RNAhybrid software [38] was used for target prediction. The sequences of the miRNAs (mature) were obtained from the miRBase database [39]. The 3’UTRs were obtained from the pacu published transcriptome [40]. We performed a target prediction between miR-1/*IGF-1*, miR-206/*IGF-1*, miR-199/*IGF-1*, miR-199/*mTOR*, miR-23a/*MAFbx*, and miR-23a/*PGC1α*. The free energy of hybridization considered was ≤ -18, providing a potential binding site through nucleotide base complementarity. The free energy observed was within the accepted ranges (S2 File).

**Fig 8:**
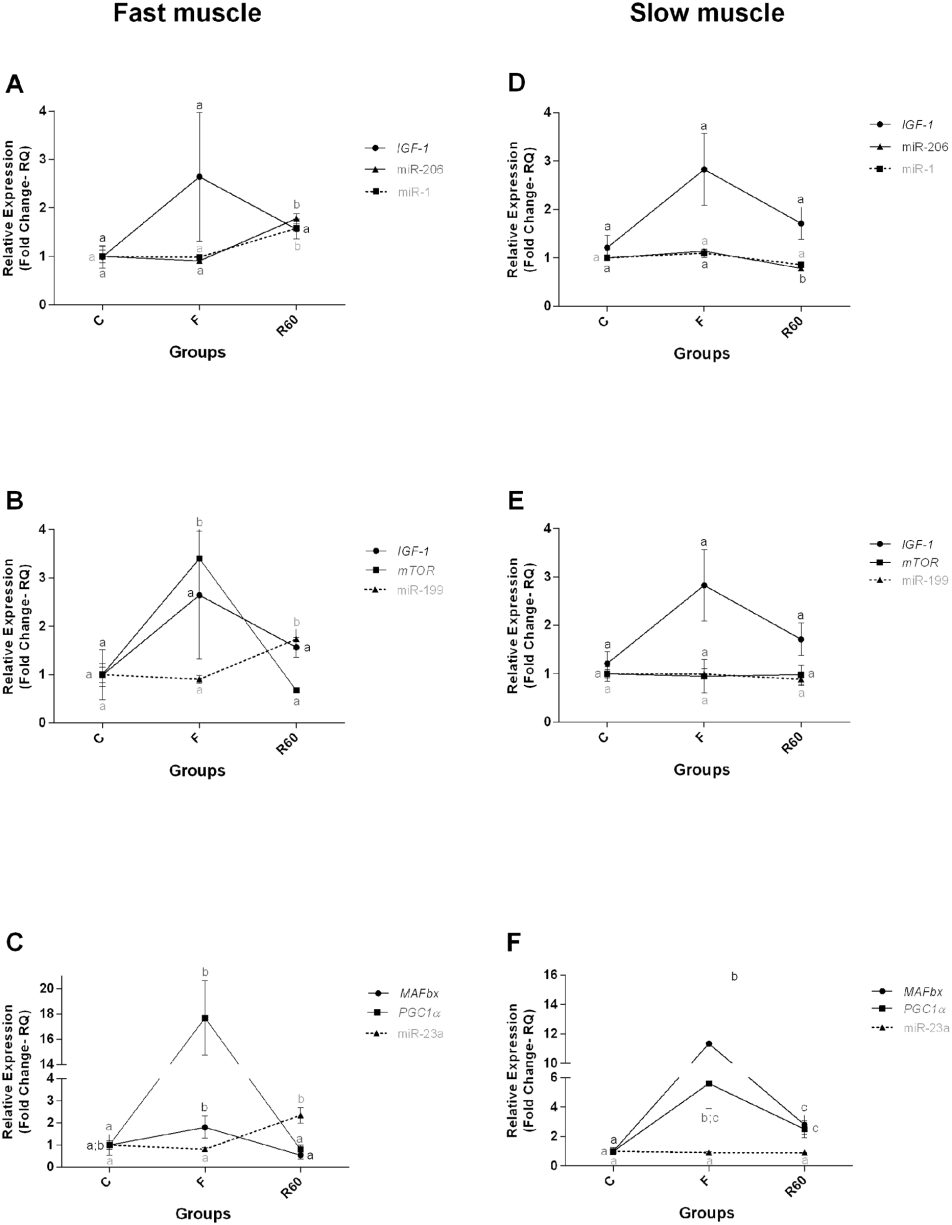
Relative expression of miRNAs and their target genes in fast and slow muscle. Groups C (control), F (fasting—10 days of fasting) and R60 (60 hours of refeeding). The data are expressed as fold change. Different letters indicate significant differences in expression between the groups (P < 0.05). The data are presented as the mean ± SEM (n = 8).

We observed changes in the expression of miRNAs that control catabolic and anabolic genes predominantly in fast muscle. The expression level of miR-1, miR-206, miR-199 and miR-23a was significantly higher in R60 than in the C and F groups in fast muscle, whereas in slow muscle, miRNAs -1 and -206 were significantly lower in R60 than in the C and F groups (Fig 8).

## Discussion

In the current study, we showed that short periods of food restriction followed by refeeding promoted changes in muscle fiber diameter, in gene expression of components involved with catabolism, anabolism and energetic metabolism and in miRNAs in both fast and slow muscle of juvenile pacu. Fast muscle was more affected in response to 10 days of food deprivation; we observed a decrease in muscle fiber diameter, an increase in the expression level of *FBXO25*, *ATG12*, *BCL2*, *PI3K*, the *mTORC1* complex (*mTOR*, *mLST8* and *RAPTOR*), *PGC1α*, *SDHA* and a decrease in *PPARβ/δA* gene expression compared with the C group. Slow muscle showed a decrease in muscle fiber diameter and slight changes in gene expression, with an increase in the levels of *MAFbx*, *PGC1α* and *PPARβ/δB* compared with the C group.

After 6 and 60 hours of refeeding, most genes were slightly up- or down-regulated in both fast and slow muscle.

Changes in muscle fiber diameter are observed during fasting conditions [41–43], and the decrease in both fast and slow muscle fiber diameter observed in our study is indicative of muscle catabolism. Additionally, based on the morphometric analysis, we can infer that after 60 hours of refeeding, only slow muscle recovered the muscle fiber area.

Muscle catabolism is regulated by several mechanisms, and the ubiquitin proteasome system (UPS) is the best-known cellular proteolytic system responsible for the degradation of the majority of cellular proteins [5,44]. The UPS system is composed of ubiquitin-activating enzymes (E1), ubiquitin carrier protein (E2) and ubiquitin-conjugating enzymes (E3 ubiquitin ligases), such as *MURF1*, and F-box proteins, such as *FBXO25* and *MAFbx*, which are responsible for muscle protein recognition, ubiquitination [45–48] and degradation [8]. Cleveland and Evenhuis [49] showed that *MAFbx* expression strongly increased in fast and slow muscle of rainbow trout (*Oncorhynchus mykiss*) after 28 days of feed deprivation, with a decreased level after refeeding periods and no alterations in *FBXO25* expression in both fast and slow muscle. Fuentes et al. [50] examined juvenile fine flounder (*Paralichthys adspersus*) after 21 days of feed deprivation and observed increased expression of *MAFbx* and *MuRF1* in fast muscle and decreased levels after four weeks of refeeding.

In our study, the high expression of *MAFbx*, *FBXO25* and *ATG12* after 10 days of fasting suggested increased muscle protein degradation, which was more intense in fast muscle. After 6 hours of refeeding, a decrease in gene expression of atrogenes in both muscles indicated strong anti-atrophic conditions during the onset of refeeding, whereas 60 hour after refeeding, red muscle presented an increased expression pattern of atrogenes (heat map analysis, S3 File).

Autophagy is an intracellular recycling system that plays important roles in the maintenance of skeletal muscle homeostasis [48,51]. During stress conditions, increased levels of autophagy are important for cells to adapt to changing nutritional and energy demands; degradation of cellular components, such as proteins, returns these materials to the cells as “building blocks” [48,51]. However, excessive activation of autophagic pathways can promote cell death [6,48,51]. *BCL2* is an anti-autophagy and anti-apoptotic protein that interacts with pro-apoptotic and pro-autophagy proteins in nutrient-limited conditions, preventing the apoptosis in muscle tissue. He et al. [52], showed that in mutant mice to *BCL2* (a model of exercise/starvation), muscle cell autophagy increased. Thus, we hypothesized that high expression of *BCL2* during feed restriction in fast muscle of pacu could represent a compensatory mechanism to prevent extreme loss of muscle mass, which was more intense in fast muscle.

Also is know that autophagy is post-translationally inhibited by the *mammalian target of rapamycin complex 1* (*mTORC1*), which is under the control of *the insulin-phosphoinositide 3-kinase* (*PI3K*)-*Akt* pathway [6,48,53,54]. Under basal conditions, *mTORC1* inhibits the protein ULK1 through selective phosphorylation. Jamart et al. [54] studied the *ULK1* complex in rats subjected to a period of fasting and exercise and observed that activation of the *ULK1* complex depends on *mTORC1* inhibition, which was also observed by Desgeorges et al. [55] in cell culture (C2C12) under starvation conditions. Thus, it is possible that the basal expression levels of *ULK1* (A and B) in fast muscle and the *ULK1B* in slow muscle during fasting in pacu are involved in *mTORC1* complex activation in this period, as noted in our experiment (heat map analysis, S3 File).

In our study, we demonstrated that the components of the *IGF1/PI3K/ Akt/mTORC1* (*mTOR*, *mLST8 and RAPTOR*) pathway were changed after food restriction and refeeding in both fast and slow muscle. *IGF-1*, a potent key regulator of muscle hypertrophy, promotes the activation of *PI3K*, which leads to the phosphorylation of the *AKT* and the *mTORC1* complex (mammalian target of rapamycin), resulting in protein synthesis [9,50,56–59].

Mareco et al. [60] described an increase in the *IGF-I* and *IGF-II* mRNA levels in the fast muscle of pacu (*Piaractus mesopotamicus*) (15 g) during fasting conditions (~ 2 days of fasting) and a decrease in the expression level during satiation feeding for 24 h. Valente et al. [61] observed in juvenile salmon (*Salmo salar* L.) after 3 weeks of fasting a difference in the expression patterns of *IGF-1* and *IGF-II*, with an increase in *IGF-1* and a decrease in *IGF-II* mRNA expression after refeeding periods. Fuentes et al. [62] also described a gradual decrease in the levels of plasma *IGF-I* in juvenile fine flounder (*Paralichthys adspersus*) during long-term of fasting (4 weeks), and following the refeeding, plasma *IGF-I* levels increased significantly.

Decreased *IGF-1* mRNA expression in starved fish muscle has been described previously [17,61,63,64], although distinct variations in the responses were observed. This fact could be related to the fasting/refeeding conditions and developmental and/or growth stage. In our experiment, increased expression levels of genes involved in the *IGF1/PI3K/ (PIK3C3)/Akt/mTORC1* pathway were observed in fast and slow muscle after 10 days of food restriction. We observed a high level of *PI3K* and *mTORC1* complex (*mTOR*, *mLST8 and RAPTOR*) expression in fast muscle, and in slow muscle, only the expression level of the subunit *mLST8* was increased in the R60 group compared with the C group.

To our knowledge, the present study demonstrates for the first time, in pacu fish, that fasting conditions increase gene expression of *mTORC1* complex in fast and slow muscle. Functional analysis in mouse and human cells culture, showed a possible physiological activation of *mTORC1* in response to feeding promoting increased expression of genes encoding proteasome subunits, increasing the intracellular pool of amino acids thus influencing the rates of protein synthesis [58]. In view of this, the results of our study indicate that possibly, as well as in mammals, the amino acids resulting through protein degradation in skeletal muscle of pacu, triggered by a stress situation (food restriction), could be important for synthesis of proteins needed for adaptation to starvation, through the activation of the *mTORC1* complex.

Metabolic adaptation is important for organism survival during starvation and long chain fatty acids, saturated and unsaturated (LCFAs), are used as a primary energy source for skeletal muscle to adapt to starvation conditions [65–68]. LCFAs regulate energy metabolism primarily by acting as an agonistic ligand of *PPARα*, *β* and *γ*, members of a nuclear receptor family of transcription factors expressed in skeletal muscle. A study using *PPARα* KO mice demonstrated a low metabolic rate and showed that these mice were unable to use fatty acids during fasting, indicating that KO mouse are reliant on protein breakdown for energy for survival; 24 h of fasting, blood urea was higher in the *PPARα* KO mice than in wild type controls, and genes for the urea cycle and protein catabolism pathways were induced in these animals [69].

In our study, the fasting period promoted an increase in the transcriptional levels of *PPARβ/δs* (A and B) in slow muscle and an abrupt decrease in fast muscle. As slow muscle has a high concentration of lipids in comparison to fast muscle [41], the high level of *PPAR* may be involved in oxidation of lipids for energy during food restriction periods [41,70]. As described by Johnston and Goldspink [70] in *Pleuronectes platessa*, during starvation, depletion of lipid reserves in the liver and muscle occurred initially, followed by muscle protein degradation. These findings can explain the higher protein breakdown in fast muscle in pacu during the fasting period.

Johnston and Goldspink [70] also described an increase in the mitochondria number in fast muscle of starved *Pleuronectes platessa*. When the rates of protein degradation are high, the transcription factor *PGC1α* (*Peroxisome proliferator-activated coactivator-1 alpha*) increases as a possible animal adaptation to an attempt to maintain the basal metabolic processes of the muscular mass [59,71–78]. We hypothesized that high expression of *PGC1α* in both fast and slow muscle in pacu after 10 days of fasting may be an adaptation to maintain the muscle metabolic demand during food restriction. The high *PGC-1α* levels (approximately 20-fold increase) in fast muscle may help remodel the muscle metabolism from glycolytic to oxidative by mitochondrial biogenesis, increasing the capacity for ATP generation (glycolytic: 2 ATP; oxidative: ~36 ATP) [4,41] during this condition, which was also confirmed by the high succinic dehydrogenase quantification.

Considering the difference between the fast and slow muscle metabolism[41] and that slow muscle shows less degradation in stress conditions, such as starvation [41], we hypothesized that the results observed in the R60 group (S3 File) could be a result of increased protein turnover [58]. However, more studies are needed to better understand the role of pathways controlling fast and slow muscle plasticity during fasting/refeeding conditions.

We also analyzed the expression of miRNAs -1, -206, -199 and -23a in fast and slow muscle and observed an inverse correlation between the expression of all miRNAs and their targets mRNAs after refeeding compared with the C and F groups in fast muscle.

Bioinformatics analysis demonstrated a possible interaction between miR-1 and the *IGF-1* gene [23]. The authors also confirmed the interaction of miR-1 and the *IGF-1* gene in cell culture and in cardiac muscle cells of animals subjected to cardiac hypertrophy, where inverse expression between miR-1 and its target, *IGF-1*, was observed. Moreover, miR-206 was also shown to regulate *IGF-1* mRNA expression, influencing muscle hypertrophy. Yan et al. [24], identified an inverse relationship between miR-206 expression and *IGF-1* mRNA expression in tilapias transfected with a miR-206 antagomir, in which fish that had a loss-of-function of this miRNA showed higher *IGF-1* mRNA expression. Shan et al. [79], using target prediction of miRNAs -1 and -206 by bioinformatics and luciferase analyses, also found an inverse relationship between the miRNAs miR-1 and miR-206 and IGF-1 protein expression in the cardiac muscle of rats with myocardial infarction.

Additionally, miR-199 also potentially regulates the *IGF-1* pathway, inhibiting the mRNA expression of *IGF-1* and *mTOR* [22]. The authors overexpressed miR-199 in cell culture (C2C12) and observed a decrease in *IGF-1* and *mTOR* expression levels; when miR-199 was knocked down, there was an increase in *IGF-1* and *mTOR* target mRNA. In our experiment, although we did not observe a statistical difference in *IGF-1* mRNA expression between the periods analyzed, the expression tended to decrease 60 hours after refeeding compared with the fasting period. We believe that the *IGF-1* expression pattern may have been influenced by the increase in miRNAs -1, -206 and -199 in fast muscle during the refeeding period. Notably, an important component of the *IGF-1* pathway, *mTOR*, had an inverse correlation with its regulator, miR-199, in both musculatures.

During the refeeding period the expression of miR-1 and miR-206 in slow muscle of pacu did not show an inverse expression pattern with *IGF-1* gene expression. As the same miRNA can regulate the expression of many different targets, and the same gene can also be regulated by more than one miRNA [80,81], we hypothesized that the decrease in miR-1 and miR-206 expression in slow muscle of pacu after refeeding may be related to the regulation of others genes that we did not analyze.

From the fasting to refeeding period, the fast muscle of juvenile pacu also showed an inverse correlation between the expression of miR-23a and the catabolic gene *MAFbx*. The increase in this miRNA is related to the translational suppression of the atrogenes *MAFbx* and *MuRF1*, as demonstrated both *in vitro*, in cell culture (C2C12) transfected with miR-23a and subjected to dexamethasone-induced atrophy, and *in vivo*, in transgenic mice expressing miR-23a and subjected to dexamethasone-induced atrophy [26]. Consistent with these results, we believe that the increase in miR-23a after refeeding in fast muscle possibly is related to a decrease in *MAFbx* expression when fast muscle catabolism decreased due to the increase of nutrients available. In addition to the interaction with *MAFbx*, miR-23a also has a role in the inhibition of *PGC-1α* [25,27] an essential cofactor of mitochondrial biogenesis [82,83]. In our study, we found an increase in *PGC-1α* gene expression during the fasting period, with a decrease after 60 hours of refeeding in both musculatures and an increase in miR-23a expression only for fast muscle. Decreased expression of miR-23a was correlated with increased *PGC-1α* in mice subjected to endurance exercise after 3 hours of activity [27]. Moreover, Russel et al. [25] also found this relationship in both cell culture (C2C12) and in transgenic mice overexpressing miR-23a. Thus, we believe that the decrease in this gene during the refeeding period was influenced by increased miR-23a in this same period in fast muscle.

Similar to the mRNA expression, fast muscle presented more differences in the miRNA expression pattern compared to slow muscle. Chu et al. [84]described, in juvenile *Siniperca chuatsi*, that several miRNAs highly expressed in fast muscle had lower expression in slow muscle and that miRNAs highly expressed in slow muscle had lower expression in fast muscle. This finding was also observed in our laboratory, where a difference in miRNA expression between fast and slow muscles in pacus during development was described [85]. Similar results were found in the present experiment, where we observed an increase in miR-1 and miR-206 expression in fast muscle and a decrease of miR-206 in slow muscle after refeeding, also described by Wiberg et al. [86] in the soleus and gastrocnemius of mice subjected to denervation.

The expression of miRNAs -23a and -199 also differed between fast and slow muscles, with increased expression in fast muscle during refeeding and no statistical differences in slow musculature. This finding highlight that in addition to more changes in mRNA expression of catabolic and anabolic genes, fast muscle also presented increased alterations in the miRNAs that control these genes. This fact may reflect that in food restriction conditions, there is a preferential utilization of fast musculature as a preferential energy source for muscle maintenance [41]. Slow muscle showed less degradation during stress conditions and, therefore, required less regulation by miRNAs.

In summary, our findings demonstrate that a short period of food restriction in juveniles pacu had a more significant effect on the fast muscle, increasing mRNA levels of anabolic (*PI3K* and mTORC1 complex: *mTOR*, *mLST8* and *RAPTOR*) and energetic metabolism genes. The miRNAs (miR-1, miR-206, miR-199 and miR-23a) were more expressed during refeeding while their target genes (*IGF-1*, *mTOR*, *PGC1α* and *MAFbx*), presented a descreased expression. We can speculate that the alterations observed in *mTORC1* complex during fasting conditions may have influenced the rates of protein synthesis by using amino acids from protein degradation as an alternative mechanism to preserve muscle phenotype and metabolic demand maintenance.

## Supporting information

S1 File miRNA binding sites at the 3'UTR of the targets mRNAs between different species of vertebrates. (TIF)

S2 File Comparative analysis of miRNA targets. (PDF)

S3 File Heat map summary and hierarchical clustering of catabolic, anabolic and cellular metabolism gene expression during fasting/refeeding condition. A: fast muscle. B: slow muscle. Red represents down-regulated genes and blue represent up-regulated genes.
(TIF)

S1 Table TaqMan^*®*^ assays used for miRNA amplification by qPCR. (DOCX)

S2 Table Primers used for selected genes and GAPDH amplification by qPCR. (DOCX)

## References

[1] Castagnolli N, Cyrino J. Piscicultura nos trópicos. 1st ed Manole; 1986.

[2] Urbinati E, Gonçalves D. Pacu (Piaractus mesopotamicus). UFSM, editor. Espécies nativas para piscicultura no Brasil. 2005.

[3] Brett J. Environmental Factors and Growth Fish Physiology. Academic Press London; 1979.

[4] Johnston I a, Bower NI, Macqueen DJ. Growth and the regulation of myotomal muscle mass in teleost fish. J Exp Biol. 2011;214: 1617–1628. 10.1242/jeb.038620 21525308

[5] Pickart CM, Eddins MJ. Ubiquitin: Structures, functions, mechanisms. Biochim Biophys Acta—Mol Cell Res. 2004;1695: 55–72. 10.1016/j.bbamcr.2004.09.019 15571809

[6] Galluzzi L, Pietrocola F, Levine B, Kroemer G. Metabolic Control of Autophagy. Cell. Elsevier Inc.; 2014;159: 1263–1276. 10.1016/j.cell.2014.11.006 PMC4500936 25480292

[7] Bonaldo P, Sandri M. Cellular and molecular mechanisms of muscle atrophy. Dis Model Mech. 2013;6: 25–39. 10.1242/dmm.010389 23268536 PMC3529336

[8] Bower NI, Johnston I a. Discovery and characterization of nutritionally regulated genes associated with muscle growth in Atlantic salmon. Physiol Genomics. 2010;42A: 114–130. 10.1152/physiolgenomics.00065.2010 20663983 PMC2957792

[9] Seiliez I, Gabillard J-C, Skiba-Cassy S, Garcia-Serrana D, Gutierrez J, Kaushik S, et al An in vivo and in vitro assessment of TOR signaling cascade in rainbow trout (Oncorhynchus mykiss). AJP Regul Integr Comp Physiol. 2008;295: R329–R335. 10.1152/ajpregu.00146.2008 18434442

[10] Cleveland BM, Weber GM. Effects of insulin-like growth factor-I, insulin, and leucine on protein turnover and ubiquitin ligase expression in rainbow trout primary myocytes. Am J Physiol Regul Integr Comp Physiol. 2010;298: R341–R350. 10.1152/ajpregu.00516.2009 20007517

[11] Fonseca R, Vabulas RM, Hartl FU, Bonhoeffer T, Nägerl UV. A Balance of Protein Synthesis and Proteasome-Dependent Degradation Determines the Maintenance of LTP. Neuron. 2006;52: 239–245. 17046687 10.1016/j.neuron.2006.08.015

[12] Suraweera A, Münch C, Hanssum A, Bertolotti A. Failure of Amino Acid Homeostasis Causes Cell Death following Proteasome Inhibition. Mol Cell. 2012;48: 242–253. 10.1016/j.molcel.2012.08.003 22959274 PMC3482661

[13] Johnston I a. Environment and plasticity of myogenesis in teleost fish. J Exp Biol. 2006;209: 2249–2264. 16731802 10.1242/jeb.02153

[14] Wullschleger S, Loewith R, Hall MN. TOR signaling in growth and metabolism. Cell. 2006;124: 471–484. 16469695 10.1016/j.cell.2006.01.016

[15] Sarbassov DD, Ali SM, Sabatini DM. Growing roles for the mTOR pathway. Curr Opin Cell Biol. 2005;17: 596–603. 16226444 10.1016/j.ceb.2005.09.009

[16] Sarbassov DD, Ali SM, Sengupta S, Sheen JH, Hsu PP, Bagley AF, et al Prolonged Rapamycin Treatment Inhibits mTORC2 Assembly and Akt/PKB. Mol Cell. 2006;22: 159–168. 16603397 10.1016/j.molcel.2006.03.029

[17] Bower NI, Li X, Taylor R, Johnston IA. Switching to fast growth: the insulin-like growth factor (IGF) system in skeletal muscle of Atlantic salmon. J Exp Biol. 2008;211: 3859–70. 10.1242/jeb.024117 19043058

[18] Byfield MP, Murray JT, Backer JM. hVps34 is a nutrient-regulated lipid kinase required for activation of p70 S6 kinase. J Biol Chem. 2005;280: 33076–33082. 16049009 10.1074/jbc.M507201200

[19] Nobukuni T, Joaquin M, Roccio M, Dann SG, Kim SY, Gulati P, et al Amino acids mediate mTOR/raptor signaling through activation of class 3 phosphatidylinositol 3OH-kinase. Proc Natl Acad Sci U S A. 2005;102: 14238–14243. 10.1073/pnas.0506925102 16176982 PMC1242323

[20] Hitachi K, Tsuchida K. Role of microRNAs in skeletal muscle hypertrophy. Front Physiol. 2014;4 1: 1–7. 10.3389/fphys.2013.00408 PMC3893574 24474938

[21] Wang XH. MicroRNA in myogenesis and muscle atrophy. Curr Opin Clin Nutr Metab Care. 2013;16: 258–266. 10.1097/MCO.0b013e32835f81b9 23449000 PMC3967234

[22] Jia L, Li YF, Wu GF, Song ZY, Lu HZ, Song CC, et al MiRNA-199a-3p regulates C2C12 myoblast differentiation through IGF-1/AKT/mTOR signal pathway. Int J Mol Sci. 2014;15: 296–308. 10.3390/ijms15010296 PMC3907811 24378853

[23] Elia L, Contu R, Quintavalle M, Varrone F, Chimenti C, Russo MA, et al Reciprocal regulation of microrna-1 and insulin-like growth factor-1 signal transduction cascade in cardiac and skeletal muscle in physiological and pathological conditions. Circulation. 2009;120: 2377–2385. 10.1161/CIRCULATIONAHA.109.879429 19933931 PMC2825656

[24] Yan B, Zhu C-D, Guo J-T, Zhao L-H, Zhao J-L. miR-206 regulates the growth of the teleost tilapia (Oreochromis niloticus) through the modulation of IGF-1 gene expression. J Exp Biol. 2013;216: 1265–9. 10.1242/jeb.079590 23197102

[25] Russell AP, Wada S, Vergani L, Hock MB, Lamon S, Léger B, et al Disruption of skeletal muscle mitochondrial network genes and miRNAs in amyotrophic lateral sclerosis. Neurobiol Dis. Elsevier Inc.; 2013;49: 107–117. 10.1016/j.nbd.2012.08.015 22975021

[26] Wada S, Kato Y, Okutsu M, Miyaki S, Suzuki K, Yan Z, et al Translational suppression of atrophic regulators by MicroRNA-23a integrates resistance to skeletal muscle atrophy. J Biol Chem. 2011;286: 38456–38465. 10.1074/jbc.M111.271270 21926429 PMC3207415

[27] Safdar A, Abadi A, Akhtar M, Hettinga BP, Tarnopolsky MA. miRNA in the regulation of skeletal muscle adaptation to acute endurance exercise in C57Bl/6J male mice. PLoS One. 2009;4: e5610 10.1371/journal.pone.0005610 19440340 PMC2680038

[28] Steven A, Bancroft J. Theory and Practice of Histological Techniques. 3rd ed New York: Churchill Livingstone; 1990.

[29] Dubowitz V, Brooke M. Muscle biopsy: A modern approach. London: WB Saunders Company; 1973.

[30] Valente LMP, Rocha E, Gomes EFS, Silva MW, Oliveira MH, Monteiro R a F, et al Growth dynamics of white and red muscle fibres in fast- and slow-growing strains of rainbow trout. J Fish Biol. 1999;55: 675–691.

[31] Johnston I. Quantitative analysis of muscle breakdown during starvation in the marine flatfish Pleuronectes platessa. Cell Tissue Res. 1981;214: 369–386. 7471184 10.1007/BF00249218

[32] de Almeida FLA, Pessotti NS, Pinhal D, Padovani CR, Leitão N de J, Carvalho RF, et al Quantitative expression of myogenic regulatory factors MyoD and myogenin in pacu (Piaractus mesopotamicus) skeletal muscle during growth. Micron. Elsevier Ltd; 2010;41: 997–1004. 10.1016/j.micron.2010.06.012 20674377

[33] Nachlas MM, Tsou K-C, De Souza E, Cheng C-S, Seligman AM. Cytochemical demonstration of succinic dehydrogenase by the use of a new p-nitrophenyl substituted ditetrazole. J Histochem Cytochem. 1957;5: 420–436. 10.1177/5.4.420 13463314

[34] Livak KJ, Schmittgen TD. Analysis of relative gene expression data using real-time quantitative PCR and. Methods. 2001;25: 402–408. 10.1006/meth.2001.1262 11846609

[35] Goodman L. On simultaneous confidence intervals for multinomial proportions. Washington: Technometrics; 1965.

[36] Zar J. Biostatistical analysis. 5rd ed New Jersey: Prentice—Hall; 2009.

[37] Kearse M, Moir R, Wilson A, Stones-Havas S, Cheung M, Sturrock S, et al Geneious Basic: An integrated and extendable desktop software platform for the organization and analysis of sequence data. Bioinformatics. Oxford University Press; 2012;28: 1647–1649. 10.1093/bioinformatics/bts199 PMC3371832 22543367

[38] Krüger J, Rehmsmeier M. RNAhybrid: MicroRNA target prediction easy, fast and flexible. Nucleic Acids Res. 2006;34: 451–454. 16845047 10.1093/nar/gkl243 PMC1538877

[39] Kozomara A, Griffiths-Jones S. MiRBase: Integrating microRNA annotation and deep-sequencing data. Nucleic Acids Res. 2011;39: 152–157. 10.1093/nar/gkq1027 PMC3013655 21037258

[40] Mareco EA, Garcia de la Serrana D, Johnston IA, Dal-Pai-Silva M. Characterization of the transcriptome of fast and slow muscle myotomal fibres in the pacu (Piaractus mesopotamicus). BMC Genomics. 2015;16: 182 10.1186/s12864-015-1423-6 25886905 PMC4372171

[41] Johnston IA. Quantitative analysis of muscle breakdown during starvation in the marine flatfish Pleuronectes platessa. Cell Tissue Res. 1981;214: 369–386. 7471184 10.1007/BF00249218

[42] Leitão N de J, Pai-Silva MD, de Almeida FLA, Portella MC. The influence of initial feeding on muscle development and growth in pacu Piaractus mesopotamicus larvae. Aquaculture. Elsevier B.V.; 2011;315: 78–85.

[43] Nebo C, Célia M, Regina F, Losi F, De Almeida A, Roberto C, et al Short periods of fasting followed by refeeding change the expression of muscle growth-related genes in juvenile Nile tilapia (Oreochromis niloticus). Comp Biochem Physiol—B Biochem Mol Biol. Elsevier B.V.; 2013;164: 268–274. 10.1016/j.cbpb.2013.02.003 23416085

[44] Komander D, Rape M. The Ubiquitin Code. Annu Rev Biochem. 2012;81: 203–229. 10.1146/annurev-biochem-060310-170328 22524316

[45] Bodine SC, Latres E, Baumhueter S, Lai VK-M, Nunez L, Clarke BA, et al Identification of Ubiquitin Ligases Required for Skeletal Muscle Atrophy. Science (80-). 2001;294. 10.1126/science.1065874 11679633

[46] LECKER SH. Multiple types of skeletal muscle atrophy involve a common program of changes in gene expression. FASEB J. 2004;18: 39–51. 10.1096/fj.03-0610com 14718385

[47] Nakashima H, Ishihara T, Suguimoto P, Yokota O, Oshima E, Kugo A, et al Chronic lithium treatment decreases tau lesions by promoting ubiquitination in a mouse model of tauopathies. Acta Neuropathol. 2005;110: 547–556. 10.1007/s00401-005-1087-4 16228182

[48] Yamano K, Matsuda N, Tanaka K. The ubiquitin signal and autophagy: an orchestrated dance leading to mitochondrial degradation. 2016;17: 300–316. 10.15252/embr.201541486 PMC4772979 26882551

[49] Cleveland BM, Evenhuis JP. Molecular characterization of atrogin-1/F-box protein-32 (FBXO32) and F-box protein-25 (FBXO25) in rainbow trout (Oncorhynchus mykiss): Expression across tissues in response to feed deprivation. Comp Biochem Physiol—B Biochem Mol Biol. Elsevier B.V.; 2010;157: 248–257. 10.1016/j.cbpb.2010.06.010 20601059

[50] Fuentes EN, Kling P, Einarsdottir IE, Alvarez M, Valdés JA, Molina A, et al Plasma leptin and growth hormone levels in the fine flounder (Paralichthys adspersus) increase gradually during fasting and decline rapidly after refeeding. Gen Comp Endocrinol. Elsevier Inc.; 2012;177: 120–127. 10.1016/j.ygcen.2012.02.019 22429729

[51] Klionsky DJ. Autophagy. Curr Biol. 2005;15: R282–R283. 10.1016/j.cub.2005.04.013 15854889

[52] He C, Bassik MC, Moresi V, Sun K, Wei Y, Zou Z, et al Exercise-induced BCL2-regulated autophagy is required for muscle glucose homeostasis. Nature. 2012;481: 511–515. 10.1038/nature10758 22258505 PMC3518436

[53] Kim J, Kundu M, Viollet B, Guan K-L. AMPK and mTOR regulate autophagy through direct phosphorylation of Ulk1. Nat Cell Biol. NIH Public Access; 2011;13: 132–141. 10.1038/ncb2152 PMC3987946 21258367

[54] Jamart C, Naslain D, Gilson H, Francaux M. Higher activation of autophagy in skeletal muscle of mice during endurance exercise in the fasted state. Am J Physiol Endocrinol Metab. 2013;305: E964–74. 10.1152/ajpendo.00270.2013 23964069

[55] Desgeorges MM, Freyssenet D, Chanon S, Castells J, Pugnière P, Béchet D, et al Post-transcriptional regulation of autophagy in C2C12 myotubes following starvation and nutrient restoration. Int J Biochem Cell Biol. Elsevier Ltd; 2014;54: 208–216. 10.1016/j.biocel.2014.07.008 25043686

[56] Fuentes EN, Einarsdottir IE, Valdes JA, Alvarez M, Molina A, Björnsson BT. Inherent growth hormone resistance in the skeletal muscle of the fine flounder is modulated by nutritional status and is characterized by high contents of truncated GHR, impairment in the JAK2/STAT5 signaling pathway, and low IGF-I expression. Endocrinology. 2012;153: 283–294. 10.1210/en.2011-1313 22028448

[57] Fuentes EN, Ruiz P, Valdes JA, Molina A. Catabolic Signaling Pathways, Atrogenes, and Ubiquitinated Proteins Are Regulated by the Nutritional Status in the Muscle of the Fine Flounder. PLoS One. 2012;7. 10.1371/journal.pone.0044256 PMC3443083 23024748

[58] Zhang Y, Nicholatos J, Dreier JR, Ricoult SJH, Widenmaier SB, Hotamisligil GS, et al Coordinated regulation of protein synthesis and degradation by mTORC1. Nature. Nature Research; 2014;513: 440–443. 10.1038/nature13492 PMC4402229 25043031

[59] Fuentes EN, Safian D, Eir I, Antonio J, Elorza AA, Molina A, et al Nutritional status modulates plasma leptin, AMPK and TOR activation, and mitochondrial biogenesis: Implications for cell metabolism and growth in skeletal muscle of the fine flounder. Gen Comp Endocrinol. Elsevier Inc.; 2013;186: 172–180. 10.1016/j.ygcen.2013.02.009 23500005

[60] Mareco EA, Garcia de la Serrana D, Johnston I a, Dal-Pai-Silva M. Characterization of the transcriptome of fast and slow muscle myotomal fibres in the pacu (Piaractus mesopotamicus). BMC Genomics. 2015;16: 182 10.1186/s12864-015-1423-6 25886905 PMC4372171

[61] Valente LMP, Bower NI, Johnston IA. Postprandial expression of growth-related genes in Atlantic salmon (Salmo salar L.) juveniles fasted for 1 week and fed a single meal to satiation. Br J Nutr. 2012;108: 2148–2157. 10.1017/S0007114512000396 22464448

[62] Fuentes EN, Björnsson BT, Valdés JA, Einarsdottir IE, Lorca B, Alvarez M, et al IGF-I/PI3K/Akt and IGF-I/MAPK/ERK pathways in vivo in skeletal muscle are regulated by nutrition and contribute to somatic growth in the fine flounder. Am J Physiol Regul Integr Comp Physiol. 2011;300: R1532–R1542. 10.1152/ajpregu.00535.2010 21389330

[63] Chauvigné F, Gabillard JC, Weil C, Rescan PY. Effect of refeeding on IGFI, IGFII, IGF receptors, FGF2, FGF6, and myostatin mRNA expression in rainbow trout myotomal muscle. Gen Comp Endocrinol. 2003;132: 209–215. 12812767 10.1016/s0016-6480(03)00081-9

[64] Terova G, Rimoldi S, Chini V, Gornati R, Bernardini G, Saroglia M. Cloning and expression analysis of insulin-like growth factor I and II in liver and muscle of sea bass (Dicentrarchus labrax, L.) during long-term fasting and refeeding. J Fish Biol. 2007;70: 219–233.

[65] Nakamura MT, Cheon Y, Li Y, Nara TY. Mechanisms of regulation of gene expression by fatty acids. Lipids. 2004;39: 1077–1083. 15726822 10.1007/s11745-004-1333-0

[66] Nakamura MT, Nara TY. STRUCTURE, FUNCTION, AND DIETARY REGULATION OF Δ6, Δ5, AND Δ9 DESATURASES. Annu Rev Nutr. Annual Reviews; 2004;24: 345–376. 10.1146/annurev.nutr.24.121803.063211 15189125

[67] Jump DB. N-3 polyunsaturated fatty acid regulation of hepatic gene transcription. Curr Opin Lipidol. NIH Public Access; 2008;19: 242–247. 10.1097/MOL.0b013e3282ffaf6a PMC2764370 18460914

[68] Nakamura MT, Yudell BE, Loor JJ. Regulation of energy metabolism by long-chain fatty acids. Prog Lipid Res. Elsevier Ltd; 2014;53: 124–144. 10.1016/j.plipres.2013.12.001 24362249

[69] Kersten S, Seydoux J, Peters JM, Gonzalez FJ, Desvergne B, Wrahli W. Peroxisome proliferator-activated receptor alpha mediates the adaptive response to fasting. J Clin Invest. 1999;103: 1489–1498. 10.1172/JCI6223 10359558 PMC408372

[70] Johnston IA, Goldspink G. Some effects of prolonged starvation on the metabolism of the red and white myotomal muscle of the plaice Pleuronectes platessa. Mar Biol. 1973;19: 348–353.

[71] Lin J, Wu H, Tarr PT, Zhang C-Y, Wu Z, Boss O, et al Transcriptional co-activator PGC-1α drives the formation of slow-twitch muscle fibres. Nature. Nature Publishing Group; 2002;418: 797–801. 10.1038/nature00904 12181572

[72] St-Pierre J, Lin J, Krauss S, Tarr PT, Yang R, Newgard CB, et al Bioenergetic analysis of peroxisome proliferator-activated receptor gamma coactivators 1alpha and 1beta (PGC-1alpha and PGC-1beta) in muscle cells. J Biol Chem. 2003;278: 26597–26603. 10.1074/jbc.M301850200 12734177

[73] Mootha VK, Handschin C, Arlow D, Xie X, St Pierre J, Sihag S, et al Errα and Gabpa/b specify PGC-1α-dependent oxidative phosphorylation gene expression that is altered in diabetic muscle. Proc Natl Acad Sci U S A. 2004;101: 6570–5. 10.1073/pnas.0401401101 15100410 PMC404086

[74] Wende AR, Huss JM, Schaeffer PJ, Gigue V, Kelly DP. PGC-1a Coactivates PDK4 Gene Expression via the Orphan Nuclear Receptor ERRa: a Mechanism for Transcriptional Control of Muscle Glucose Metabolism. Mol Cell Biol. 2005;25: 10684–10694. 10.1128/MCB.25.24.10684-10694.2005 16314495 PMC1316952

[75] Sandri M, Lin J, Handschin C, Yang W, Arany ZP, Lecker SH, et al PGC-1alpha protects skeletal muscle from atrophy by suppressing FoxO3 action and atrophy-specific gene transcription. Proc Natl Acad Sci U S A. 2006;103: 16260–5. 10.1073/pnas.0607795103 17053067 PMC1637570

[76] Wende AR, Schaeffer PJ, Parker GJ, Zechner C, Han DH, Chen MM, et al A role for the transcriptional coactivator PGC-1α in muscle refueling. J Biol Chem. 2007;282: 36642–36651. 10.1074/jbc.M707006200 17932032

[77] Vechetti IJ, Bertaglia RS, Fernandez GJ, De Paula TG, De Souza RWA, Moraes LN, et al Aerobic Exercise Recovers Disuse-induced Atrophy Through the Stimulus of the LRP130/PGC-1α Complex in Aged Rats. Journals Gerontol—Ser A Biol Sci Med Sci. 2016;71: 601–609. 10.1093/gerona/glv064 25991827

[78] Hoppeler H. Molecular networks in skeletal muscle plasticity. J Exp Biol. 2016;219: 205–13. 10.1242/jeb.128207 26792332

[79] Shan ZX, Lin QX, Fu YH, Deng CY, Zhou ZL, Zhu JN, et al Upregulated expression of miR-1/miR-206 in a rat model of myocardial infarction. Biochem Biophys Res Commun. Elsevier Inc.; 2009;381: 597–601. 10.1016/j.bbrc.2009.02.097 19245789

[80] Stark A, Brennecke J, Bushati N, Russell RB, Cohen SM. Animal MicroRNAs Confer Robustness to Gene Expression and Have a Significant Impact on 3′UTR Evolution. Cell. 2005;123: 1133–1146. 10.1016/j.cell.2005.11.023 16337999

[81] van Rooij E, Liu N, Olson EN. MicroRNAs flex their muscles. Trends Genet. 2008;24: 159–166. 10.1016/j.tig.2008.01.007 18325627

[82] Puigserver P, Rhee J, Donovan J, Walkey CJ, Yoon JC, Oriente F, et al Insulin-regulated hepatic gluconeogenesis through FOXO1–PGC-1α interaction. Nature. 2003;423: 550–555. 10.1038/nature01667 12754525

[83] Schiaffino S, Dyar KA, Ciciliot S, Blaauw B, Sandri M. Mechanisms regulating skeletal muscle growth and atrophy. FEBS J. 2013;280: 4294–4314. 10.1111/febs.12253 23517348

[84] Chu W-Y, Liu L-S, Li Y-L, Chen L, Wang K-Z, Li H-H, et al Systematic identification and differential expression profiling of MicroRNAs from white and red muscles of siniperca chuatsi. Curr Mol Med. 2013;13: 1397–407. Available: http://www.ncbi.nlm.nih.gov/pubmed/23826919 23826919 10.2174/15665240113139990059

[85] Duran BODS, Fernandez GJ, Mareco EA, Moraes LN, Salomão RAS, Gutierrez De Paula T, et al Differential microRNA expression in fast- and slow-twitch skeletal muscle of Piaractus mesopotamicus during growth. PLoS One. 2015;10: 1–16. 10.1371/journal.pone.0141967 PMC4631509 26529415

[86] Wiberg R, Jonsson S, Novikova LN, Kingham PJ. Investigation of the expression of myogenic transcription factors, microRNAs and muscle-specific E3 ubiquitin ligases in the medial gastrocnemius and soleus muscles following peripheral nerve injury. PLoS One. 2015;10: 1–20. 10.1371/journal.pone.0142699 PMC4686181 26691660

