# Supplementary figures and images for: Food restriction increase the expression of mTORC1 complex genes in the skeletal muscle of juvenile pacu (Piaractus mesopotamicus)
Source: PLoS One. 2017 May 15;12(5):e0177679. doi: 10.1371/journal.pone.0177679 (PMC5432107; doi:10.1371/journal.pone.0177679)

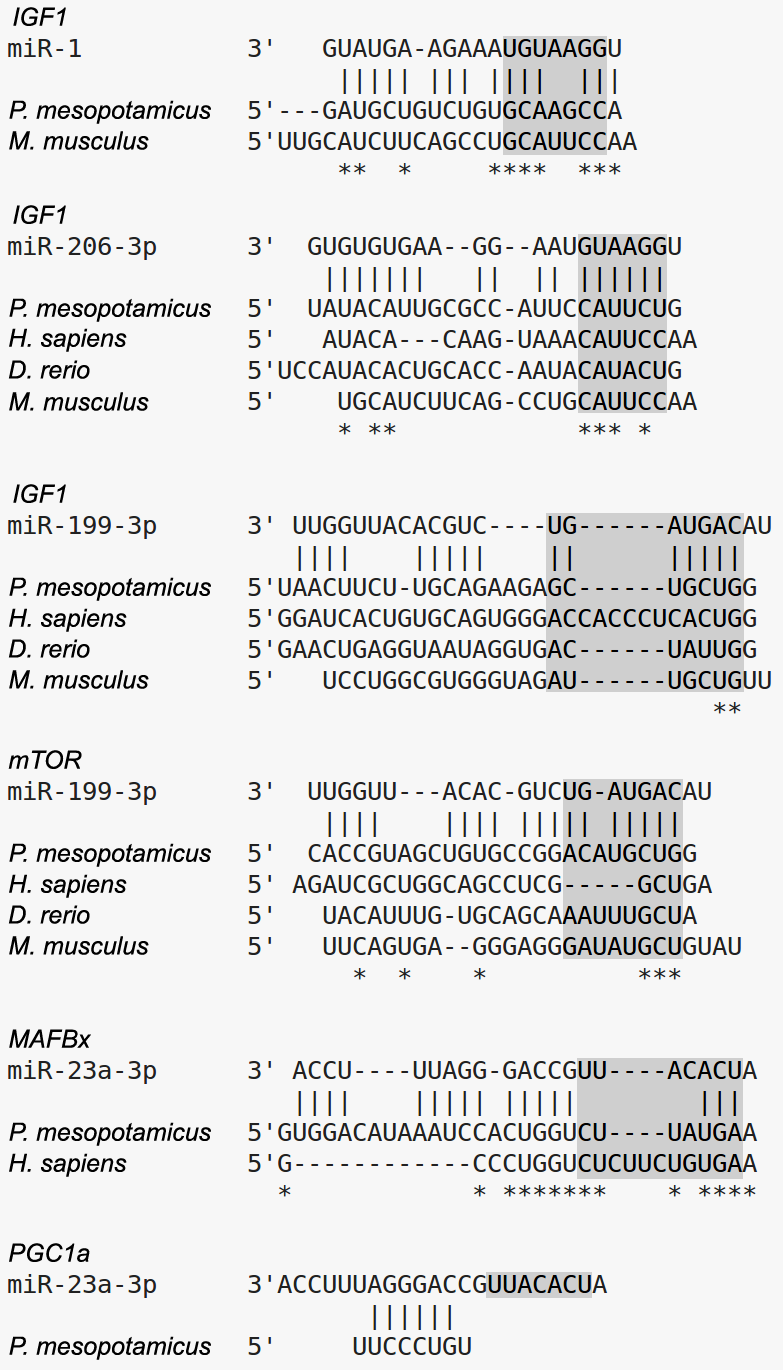

Supplement: S1 File — (TIF) [file pone.0177679.s001.tif]

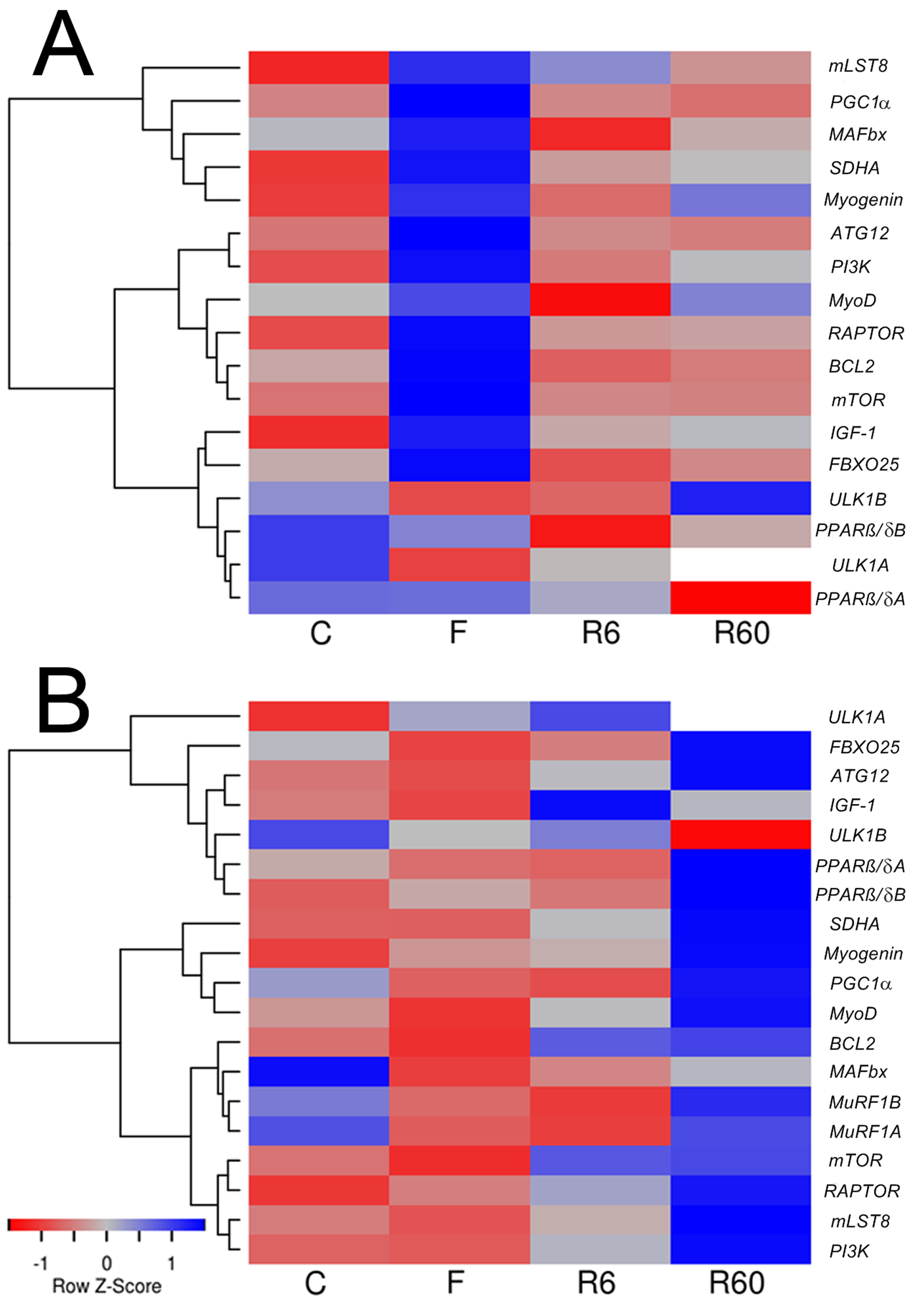

Supplement: S3 File — A: fast muscle. B: slow muscle. Red represents down-regulated genes and blue represent up-regulated genes. (TIF) [file pone.0177679.s003.tif]
