# Supplementary material for: Food restriction increase the expression of mTORC1 complex genes in the skeletal muscle of juvenile pacu (Piaractus mesopotamicus)
Source: PLoS One. 2017 May 15;12(5):e0177679. doi: 10.1371/journal.pone.0177679 (PMC5432107; doi:10.1371/journal.pone.0177679)

Comparative analysis of miRNA targets

```
Version: RNAhybrid 2.2

searching
dataset: 1
mde of dre-miR-1: -38.299995
Individual hits
```

dataset: 1
Target: *utr3_IGF1_Piaractus_mesopotamicus*
length: 700
MiRNA: *dre-miR-1*
length: 22

mfe: -18.3 kcal/mol
p-value: undefined

Position: 535

```
target 5'    G     G   G    AG   U 3'
              AUGCU UCU UGCA  CCA
              UAUGA AGA AUGU  GGU
miRNA  3' UAUG          A    AA     5'
```

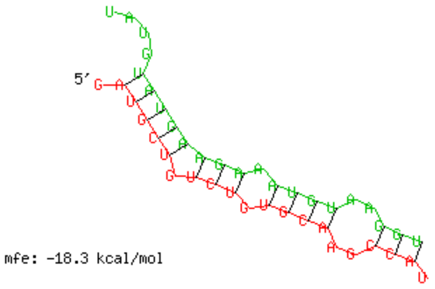


```
Version: RNAhybrid 2.2

searching
dataset: 1
mde of dre-miR-206-3p: -45.699997
Individual hits
```

dataset: 1
Target: *utr3_IGF1_Piaractus_mesopotamicus*
length: 700
MiRNA: *dre-miR-206-3p*
length: 22

mfe: -20.8 kcal/mol
p-value: undefined

Position: 183

```
target 5' U        GCG  A  C      G 3'
           UAUACAUU   CC UU CAUUCU
           GUGUGUGA   GG AA GUAAGG
miRNA  3' G        A       U      U 5'
```

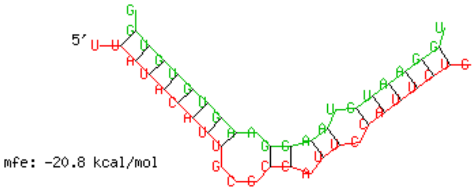


```
Version: RNAhybrid 2.2

searching
dataset: 1
mde of dre-miR-199-3p: -44.100002
Individual hits
```

dataset: 1
Target: *utr3_IGF1_Piaractus_mesopotamicus*
length: 700
MiRNA: *dre-miR-199-3p*
length: 22

mfe: -22.4 kcal/mol
p-value: undefined

Position: 306

```
target 5' U    UCU     AAGA       G  3'
           AACU    UGCAG    GCUGCUG
           UUGG    ACGUC    UGAUGAC
miRNA  3'     UUAC                AU 5'
```

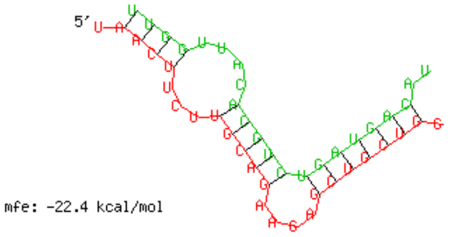

```
Version: RNAhybrid 2.2

searching
dataset: 1
mde of dre-miR-199-3p: -44.100002
Individual hits
```

---

dataset: 1
Target: *utr3_mTOR_Piaractus_mesopotamicus*
length: 1022
MiRNA: *dre-miR-199-3p*
length: 22

mfe: -25.8 kcal/mol
p-value: undefined

Position: 902

```
target 5' C     UAGC     C      A      G   3'
           ACCG     UGUG CGGAC UGCUG
           UGGU     ACAC GUCUG AUGAC
miRNA  3' U     U                     AU 5'
```

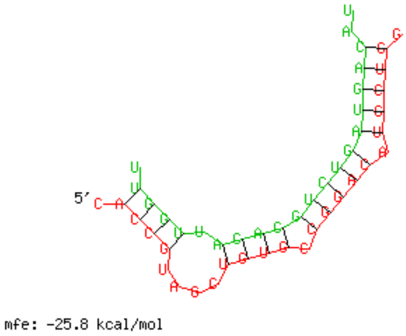

```
Version: RNAhybrid 2.2

searching
dataset: 1
mde of dre-miR-23a-3p: -46.299995
Individual hits
```

---

dataset: 1
Target: *utr3_MAFBx_Piaractus_mesopotamicus*
length: 537
MiRNA: *dre-miR-23a-3p*
length: 22

mfe: -23.1 kcal/mol
p-value: undefined

Position: 197

```
target 5' G     CAUA      A      CUUUA    A 3'
           UGGA     AAUCC CUGGU      UGA
           ACCU     UUAGG GACCG      ACU
miRNA  3'                      UUAC     A 5'
```

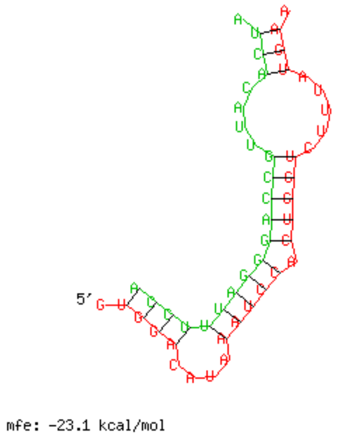

```
Version: RNAhybrid 2.2

searching
dataset: 1
mde of dre-miR-23a-3p: -46.299995
Individual hits
```

---

dataset: 1
Target: *utr3_PGC1a_Piaractus_mesopotamicus*
length: 202
MiRNA: *dre-miR-23a-3p*
length: 22

mfe: -14.9 kcal/mol
p-value: undefined

Position: 149

```
target 5'       U      U           3'
                 UCCCUG
                 AGGGAC
miRNA  3' ACCUUU      CGUUACACUA 5'
```

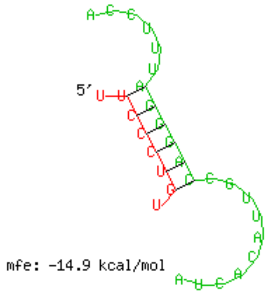

Supplement: S2 File — (PDF) [file pone.0177679.s002.pdf]
