# Supplementary material for: Food restriction increase the expression of mTORC1 complex genes in the skeletal muscle of juvenile pacu (Piaractus mesopotamicus)
Source: PLoS One. 2017 May 15;12(5):e0177679. doi: 10.1371/journal.pone.0177679 (PMC5432107; doi:10.1371/journal.pone.0177679)
Supplement: S1 Table — (DOCX) [file pone.0177679.s004.docx]

**S1 Table.** TaqMan*®* assays used for miRNA amplification by qPCR.

| **miRBase ID** | **miRBase accession number** | **Target sequence** |
| --- | --- | --- |
| **dre-miR-1** | MIMAT0001768 | UGGAAUGUAAAGAAGUAUGUAU |
| **dre-miR-206-3p** | MIMAT0001866 | UGGAAUGUAAGGAAGUGUGUGG |
| **dre-miR-23a-3p** | MIMAT0001790 | AUCACAUUGCCAGGGAUUUCCA |
| **dre-miR-199-3p** | MIMAT0003155 | UACAGUAGUCUGCACAUUGGUU |
| **U6 snRNA** | Acess number NCBI: NR_004394 | |
