# Supplementary material for: Food restriction increase the expression of mTORC1 complex genes in the skeletal muscle of juvenile pacu (Piaractus mesopotamicus)
Source: PLoS One. 2017 May 15;12(5):e0177679. doi: 10.1371/journal.pone.0177679 (PMC5432107; doi:10.1371/journal.pone.0177679)
Supplement: S2 Table — (DOCX) [file pone.0177679.s005.docx]

**S2 Table.** Primers used for selected genes and *GAPDH* amplification by qPCR.

| **Gene** | **Primer sequences (5’ → 3’)** | **Size of amplified fragment (bp)** | |
| --- | --- | --- | --- |
| ***ATG12*** | L: AGTTCATCTCCCGCTTCCTT | 120 | |
|  | R: CACTGCCGAAACACTCAAAA |  |  |
| ***BCL2*** | L: CCCCTCCTCTCTCTTCGTCT | 135 | |
|  | R: TGTCCATTTCCTGCTGTTTG |  |  |
| ***ULK1A*** | L: GACAAGCCAACCAGGAGAGT  R:GGTAGTTGTGGATTTGTGTA | 192 | |
| ***ULK1B*** | L: CTTTGAGCTGTGGCAGGACA  R:CTTGTGGGGAGAGCTGGAAG | 105 | |
| ***FBXO25*** | L: CGTAGGTGAAGTAGCCGCCA | 166 | |
|  | R: GGAGAAGTCCAGCCTGTTGA |  |  |
| ***MAFbx*** | L: TCTTTGGTGCTCCCCTTGTG | 231 | |
|  | R: TAAAACCGAGGACGGCTGG |  |  |
| ***MURF1A*** | L: CCATTGATGCCTTGGTTGC | 198 | |
|  | R: CCTTCTCGTCCTGCTCTTTG |  |  |
| ***MURF1B*** | L: CCCCCTGAAAGACAAAGACA | 179 | |
|  | A: GCTCAGCCTTCTGGACTTC |  |  |
| ***IGF-1*** | L: ATTTCAGCAAGCCAACAGGT | 116 | |
|  | R: CGCACAATACATCTCAAGTCG |  |  |
| ***PI3K*** | L: GTCTGGAGCCTGGCAGTAAG | 119 | |
|  | R: CTTCTGTGTGCTGGGAGTCA |  |  |
| ***mTOR*** | L: TTGGGAGAGACGTACTGC  R: CACAGGACTGGTGTAGGAA | 145 | |
| ***RAPTOR*** | L: TGTTTACGACTGCTCCAA  R: GAGGGTGGTTCGGGTTTATT | 107 | |
| ***GBL***  ***(mLST8)*** | L: TCCCAAGACCAAAATTCCTG  R: AAAGTTTGAGGTTCGCCAGA | 124 | |
| ***SDHA*** | L: ACCTGATGCTGAATGCTGTG  R: AGTGTGCTTCCTCCAGTGC | 170 | |
| ***PGC1α*** | L: GAGGGTGAGCGTTCAAAGAG  R: ATGAGGCTGAGCAGAGAGGA | | 187 |
| ***MyoD*** | L: GTTCGTCGTCTTCCTCTTGC  R: ACCCGTGCTTTAACACCAAC | | 191 |
| ***Myogenin*** | L: CAGACCAGAGGTTTTATGAA  R: TAGATGTTGGGGATGGCTTG | | 171 |
| ***PPARβ/δA*** | L: TAGCCACACCTCCTCCCTAA  R: GCTGCTGTTCTCCTCCAATC | | 136 |
| ***PPARβ/δB*** | L: GAAGGAGAAGGAAGCGAGGT  R:GTCAACTTCATCCCCATTCT | | 102 |
| ***GAPDH*** | L: ACACACGACGACAAGACCAA  R: GTCCCTCTCGCTGAAAACTG | | 267 |
